# The novel ciliogenesis regulator DYRK2 governs Hedgehog signaling during mouse embryogenesis

**DOI:** 10.7554/eLife.57381

**Published:** 2020-08-06

**Authors:** Saishu Yoshida, Katsuhiko Aoki, Ken Fujiwara, Takashi Nakakura, Akira Kawamura, Kohji Yamada, Masaya Ono, Satomi Yogosawa, Kiyotsugu Yoshida

**Affiliations:** 1 Department of Biochemistry, The Jikei University School of MedicineTokyoJapan; 2 Division of Histology and Cell Biology, Department of Anatomy, Jichi Medical University School of MedicineTochigiJapan; 3 Department of Anatomy, Graduate School of Medicine, Teikyo UniversityTokyoJapan; 4 Department of Clinical Proteomics, National Cancer Center Research InstituteTokyoJapan; University of CopenhagenDenmark; Brandeis UniversityUnited States

**Keywords:** DYRK2, ciliogenesis, hedgehog signal, development, Mouse

## Abstract

Mammalian Hedgehog (Hh) signaling plays key roles in embryogenesis and uniquely requires primary cilia. Functional analyses of several ciliogenesis-related genes led to the discovery of the developmental diseases known as ciliopathies. Hence, identification of mammalian factors that regulate ciliogenesis can provide insight into the molecular mechanisms of embryogenesis and ciliopathy. Here, we demonstrate that DYRK2 acts as a novel mammalian ciliogenesis-related protein kinase. Loss of *Dyrk2* in mice causes suppression of Hh signaling and results in skeletal abnormalities during in vivo embryogenesis. Deletion of *Dyrk2* induces abnormal ciliary morphology and trafficking of Hh pathway components. Mechanistically, transcriptome analyses demonstrate down-regulation of *Aurka* and other disassembly genes following *Dyrk2* deletion. Taken together, the present study demonstrates for the first time that DYRK2 controls ciliogenesis and is necessary for Hh signaling during mammalian development.

## Introduction

Embryogenesis and patterning of cell differentiation are facilitated by spatiotemporal activation of multiple signaling pathways. The Hedgehog (Hh) signaling is an evolutionarily conserved system that plays a central role in embryogenesis via regulating cell proliferation and differentiation (Ingham and McMahon, 2001). Upon stimulation by ligands, post-translational modification of GLI2 and GLI3 induces the expression of *Gli1*, which is a key amplifier of Hh signaling. These post-translational and transcriptional activations of three GLIs regulate specific and redundant target genes (Mo et al., 1997; Hui and Angers, 2011). Hence, mutants of Hh components cause typical defects such as skeletal, neural, and retinal abnormalities (Mo et al., 1997).

Unlike other core developmental signaling, vertebrate Hh signaling is uniquely and completely dependent upon primary cilia, which are microtubule-based organelles that are formed during the G_0_ or G_1_ phases of the cell cycle (Huangfu et al., 2003). Binding of Hh ligands to Patched 1 (PTCH1) on cilia leads to activation and induction of Seven-spanner smoothened (SMO) to the cilia (Rohatgi et al., 2007). Activated SMO leads to recruitment of GLI2 and GLI3 to the cilia tip via inhibition of protein kinase A (Chen et al., 2009; Kim et al., 2009; Wen et al., 2010). This dynamic ciliary trafficking of Hh components is primarily regulated by intraflagellar transport (IFT) (Haycraft et al., 2005; Eguether et al., 2014). Thus, ciliogenesis is indispensable for tissue development, and defects in this process impact the development of multiple organs to cause human and mouse diseases termed ‘ciliopathies’ (Reiter and Leroux, 2017). Accordingly, the typical phenotype observed in some of ciliopathies such as Joubert syndrome is abnormalities of Hh signaling (Bangs and Anderson, 2017).

Mutations in a number of different ciliopathy-associated genes often result in alterations of ciliary length (Paige Taylor et al., 2016; Reiter and Leroux, 2017). Indeed, genetic screenings of *Chlamydomonas*, which is a model organism for ciliogenesis, have identified the ciliopathy-associated genes controlling cilia length to generate the optimal length at steady-state (Wemmer and Marshall, 2007). Although these abnormalities in ciliary length are thought to be controlled by the balance of assembly and disassembly via IFT and a postulated length sensor, the mechanisms for maintaining the cell-type-specific ciliary length have not been fully elucidated (Ishikawa and Marshall, 2011). In contrast, the ciliary resorption mechanisms for cell cycle re-entry have been thoroughly investigated by such as an experiment of serum re-addition to starved cells, and these mechanisms include the HEF1-AURKA-HDAC6 pathway (Pugacheva et al., 2007), the PLK-KIF2A pathway (Wang et al., 2013), and the NEK2-KIF24 pathway (Kobayashi et al., 2011; Kim et al., 2015b) that have been observed to induce disassembly of cilia. On the other hand, the ability of these ciliary resorption factors for cell cycle re-entry to control cilia length at steady-state and during ciliogenesis remains to be elucidated. Hence, identification of novel mammalian factors regulating ciliogenesis and ciliary length control will provide insight into the molecular mechanisms underlying embryogenesis and ciliopathy as well as ciliary functions.

Dual-specificity tyrosine-regulated kinase (DYRK) is a family that belongs to the CMGC group that includes cyclin-dependent kinases (CDK), mitogen-activated protein kinase (MAPK), glycogen synthase kinase (GSK), and CDK-like kinase (CLKs) (Becker and Sippl, 2011). Two isoforms of *Dyrk2* have been identified; long and short forms, the latter lacks a 5’ terminal region. In human cancer cells, we have functionally identified DYRK2 as a regulator of p53-induced apoptosis in response to DNA damage (Taira et al., 2007) and of G1/S transition (Taira et al., 2012). During development in lower eukaryotes, MBK2, which is an ortholog of DYRK2 in *Caenorhabditis elegans*, regulates maternal-protein degradation during the oocyte-to-embryo transition via a ubiquitin-dependent mechanism (Pellettieri et al., 2003; Pang et al., 2004; Lu and Mains, 2007; Yoshida and Yoshida, 2019). While these reports lead us to speculate that DYRK2 must also play important roles in mammalian development, no reports are available regarding the mechanistic role of DYRK2 in vivo.

In the present study, we aim to reveal a function for DYRK2 in mammalian development in vivo. Here, we demonstrate that DYRK2 is a novel regulator of ciliogenesis and is required for normal embryogenesis via activation of Hh signaling during development.

## Results

### *Dyrk2* deficiency cause suppression of Hedgehog signaling during mouse embryogenesis

**Figure 1.**
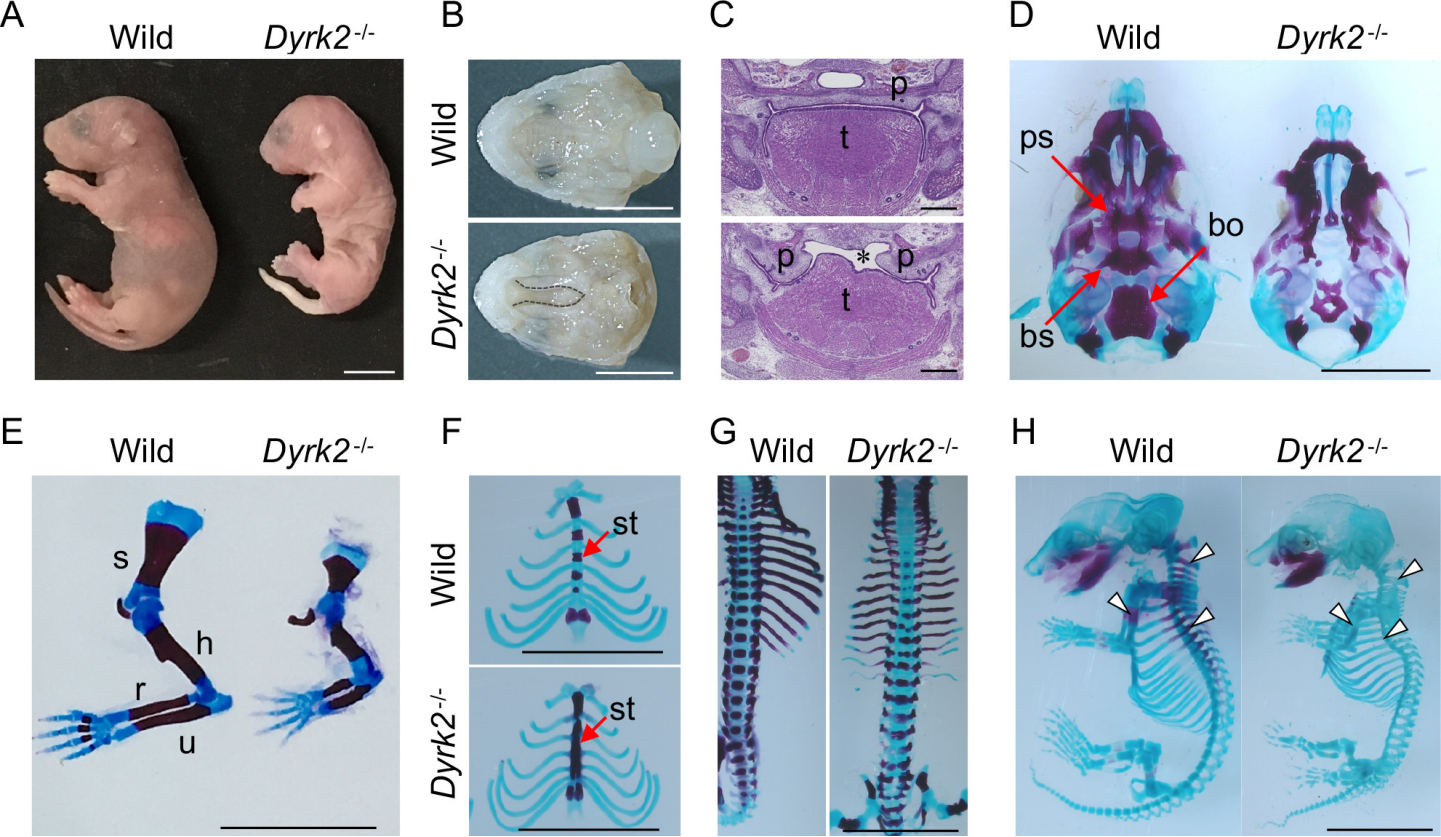
Deletion of DYRK2 shows skeletal defects in mouse development. (**A**) Whole embryo gross images of wild-type and homozygous *Dyrk2^-/-^* embryos at birth. (**B, C**) Palatal and tongue abnormalities in *Dyrk2^-/-^* embryos. Gross images of the palate with mandible removed from wild-type and *Dyrk2^-/-^* embryos at E18.5 (**B**), and HE staining from the coronal plane at E13.5 (**C**). Dotted lines in (**B**) and an asterisk in (**C**) indicate cleft of the secondary palate. (**D–H**) Arizarin red and alcian blue staining of the craniofacial skeleton (**D**), forelimbs (**E**), sternum (**F**), and vertebra (**G**) from wild-type and *Dyrk2^-/-^* embryos at E18.5, and whole skeleton staining at E16.5 (**H**). Arrowheads in (**H**) indicate regions that decreasing bone mineralization. bo, basioccipital bone; bs, basisphenoid; h, humerus; r, radius; p, palatal shelves; ps, presphenoid; s, scapula; st, sternebrae; t, tongue; u, ulna. Scale bars, 5 mm.

We generated *Dyrk2* knockout mice (*Dyrk2^-/-^*) by eliminating the third exon of the *Dyrk2* genomic locus (Figure 1—figure supplement 1A–B). The absence of DYRK2 protein in homozygous *Dyrk2^-/-^* mice was confirmed (Figure 1—figure supplement 1C). Although the gross morphology of homozygous *Dyrk2^-/-^* embryos appeared normal during early development, multiple defects became obvious during later stages of gestation, and the mice died at or close to birth (Figure 1A). Specifically, defects in skeletal development were remarkable, and these included a shorter dorsum of the nose (Figure 1A), cleft palate including hypoplasia of the tongue (Figure 1B–C), loss of the basisphenoid, basioccipital, and presphenoid bones (Figure 1D), shorter limbs (Figure 1E), defects of segmentation of the sternebrae in the sternum (Figure 1F), and vertebra (Figure 1G) at embryonic day (E) 18.5. These skeletal defects that included reduction of bone mineralization were observed until E16.5 (Figure 1H).

**Figure 1—figure supplement 1.**
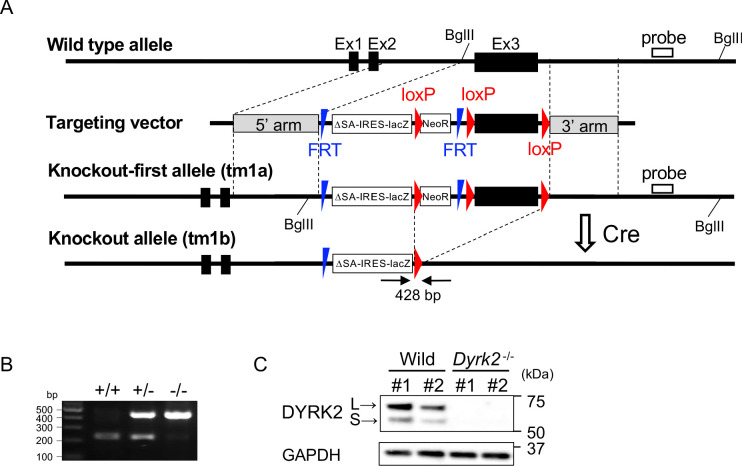
Generation of *Dyrk2^-/-^* mice schematic representation of the *Dyrk2^-/-^* allele (*Dyrk2*^tm1b^). (**A**) Cre-mediated recombination was used to generate the *Dyrk2^-/-^* allele (*Dyrk2*^tm1b^) from the floxed allele (*Dyrk2*^tm1a^). The black boxes, red arrowheads, and blue arrowheads indicate exons, *loxP* sites, and *FRT* sites, respectively. (**B**) PCR-confirmed mutagenesis. (**C**) Immunoblotting of DYRK2 in extracts from each wild-type and *Dyrk2^-/-^* embryo at E13.5. L and S indicate long and short transcriptional isoforms of DYRK2, respectively.

**Figure 2.**
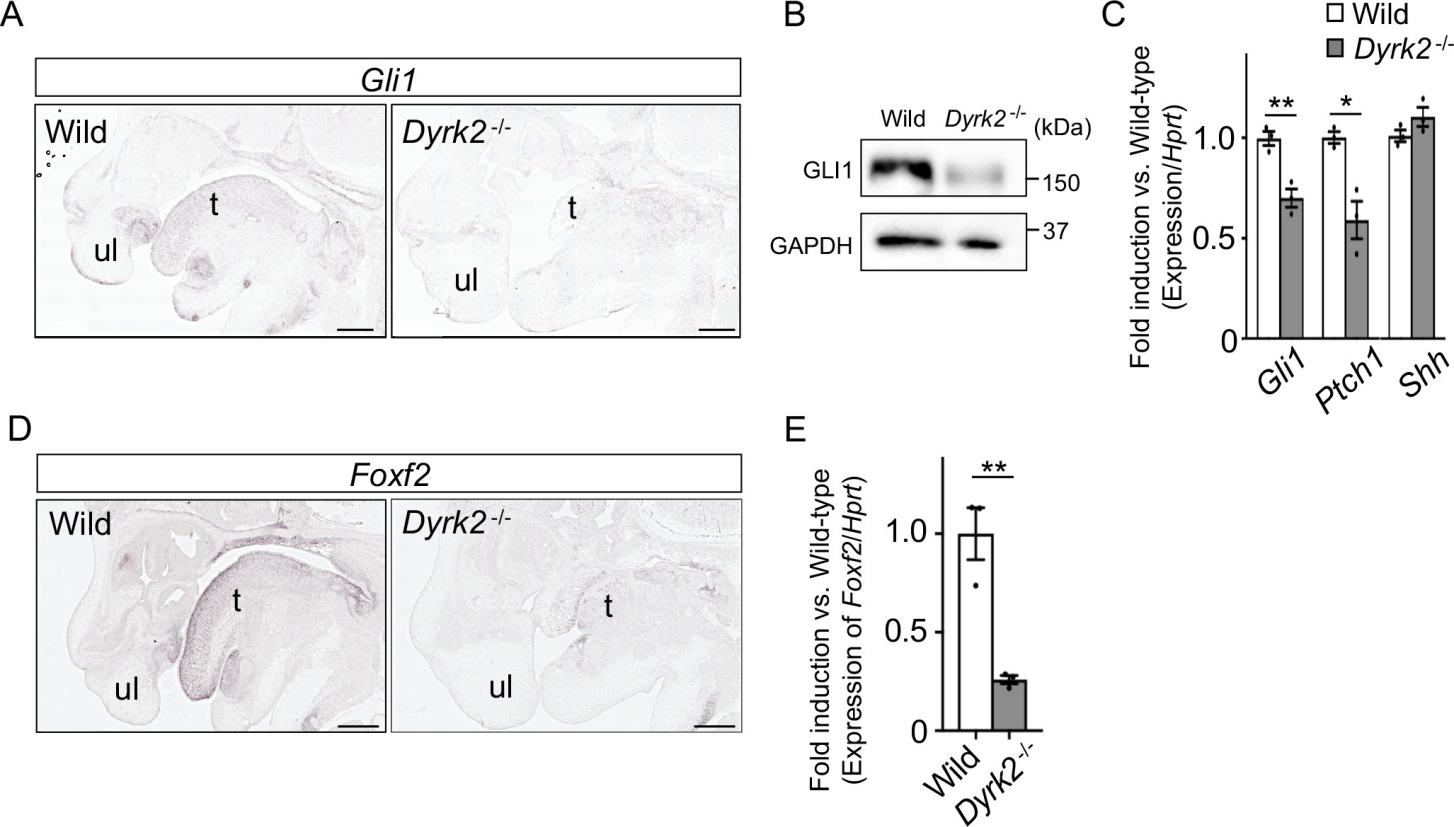
Deletion of DYRK2 affects activation of Hh signaling in mouse development. (**A**) In situ hybridization of *Gli1* in the craniofacial region in wild-type and *Dyrk2^-/-^* embryos from the sagittal plane at E14.5. (**B**) Immunoblotting of GLI1 in extracts from the limbs of wild-type and *Dyrk2^-/-^* embryos at E13.5. GAPDH serves as a loading control. (**C**) qPCR of *Gli1*, *Ptch1*, and *Shh* in the limbs from wild-type and *Dyrk2^-/-^* embryos at E13.5. (**D, E**) Repression of *Foxf2*-expression in the craniofacial region of *Dyrk2^-/-^* mice. (**D**) In situ hybridization of *Foxf2* in the craniofacial region in wild-type and *Dyrk2^-/-^* embryos from the sagittal plane at E14.5. (**E**) qPCR of *Foxf2* in the mandibular arch from wild-type and *Dyrk2^-/-^* embryos at E10.5. Hypoxanthine phosphoribosyltransferase (*Hprt*) in (**C and E**) was used as an internal standard, and fold change was calculated by comparing expression levels relative to those of wild-type. Data are presented as the means ± SEM (*n* = 3 biological replicates). The statistical significance between wild-type and *Dyrk2^-/-^* was determined by the Student’s *t*-test. (*) p<0.05, (**) p<0.01. t, tongue; ul, upper lip. Scale bars, 500 µm. Figure 2—source data 1.Source data for Figure 2C and E.

As *Dyrk2^-/-^* embryos at E18.5 exhibited a similar phenotype to that observed in response to certain defects in Hh signaling (Mo et al., 1997), we assessed *Gli1*-expression, which is an indicator of Hh signaling activation (Niewiadomski and Rohatgi, 2015). In situ hybridization demonstrated that *Gli1*-expression was decreased in the craniofacial region in *Dyrk2^-/-^* embryos at E14.5 (Figure 2A). Protein levels of GLI1 were also decreased at E13.5 (Figure 2B). *Ptch1*-expression, which is another indicator of Hh signaling activation (Snouffer et al., 2017), was also decreased in *Dyrk2^-/-^* embryos at E13.5, and this was accompanied by a decrease in *Gli1*; however, *Shh*-expression remained unchanged (Figure 2C). We also observed a repression of *Foxf2*-expression, which is a direct target gene of GLI1 (Everson et al., 2017), in the craniofacial region of *Dyrk2^-/-^* embryos (Figure 2D–E).

**Figure 2—figure supplement 1.**
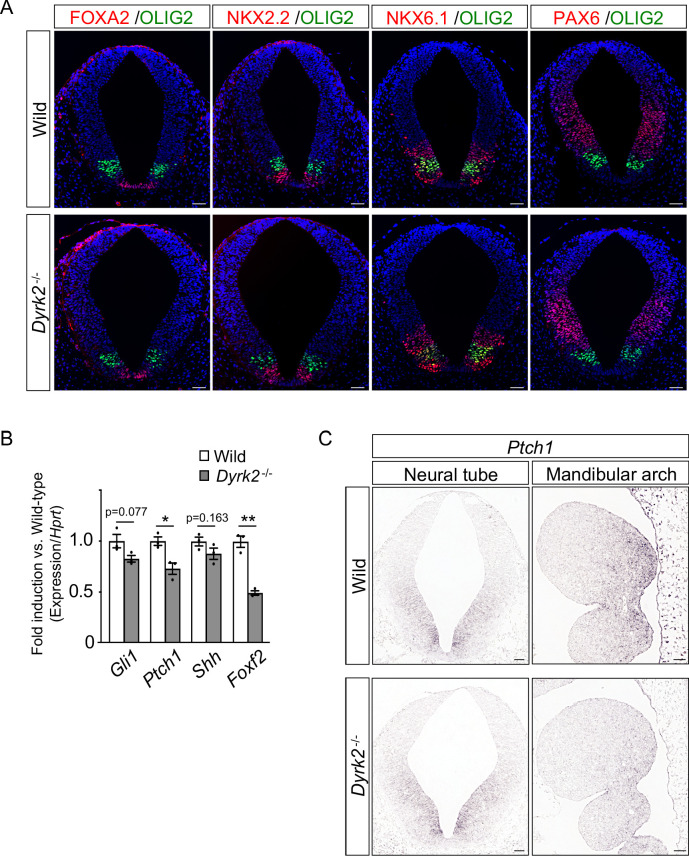
Dorsal-ventral patterning of the neural tube in *Dyrk2^-/-^* mice. (**A**) Transverse sections of wild-type and *Dyrk2^-/-^* embryos at E10.5 (at the branchial level) were stained for markers of ventral (FOXA2, NKX2.2, OLIG2, and NKX6.1) and dorsal (PAX6) regions. Nuclei were stained with DAPI (blue). (**B**) qPCR of *Gli1*, *Ptch1*, *Shh*, and *Foxf2* in the whole embryos from wild-type and *Dyrk2^-/-^* embryos at E9.5. Data are presented as the means ± SEM (*n* = 3 biological replicates). The statistical significance between wild-type and *Dyrk2^-/-^* was determined by the Student’s *t*-test. (*) p<0.05, (**) p<0.01. (**C**) In situ hybridization of *Ptch1* in the neural tube (left panels) and mandibular arch (right panels) in wild-type and *Dyrk2^-/-^* embryos at E10.5 from the transverse and sagittal plane, respectively. Scale bars, 50 µm. Figure 2—figure supplement 1—source data 1.Source data for Figure 2—figure supplement 1B.

Loss of genes required for Hh signaling often causes defects in dorsal-ventral neural tube patterning, which is regulated by the SHH morphogen (Dessaud et al., 2008). Although we investigated the localization patterns of FOXA2, NKX2.2, OLIG2, NKX6.1, and PAX6 at E10.5, we did not observe obvious differences in their expression patterns (Figure 2—figure supplement 1A). Expression of Hh target genes was decreased in *Dyrk2^-/-^* whole embryos at E9.5 (Figure 2—figure supplement 1B). In situ hybridization demonstrated that *Ptch1*-expression was decreased in the mandibular arch in *Dyrk2^-/-^* embryos at E10.5, but remained unchanged in the neural tube (Figure 2—figure supplement 1C). These data showing the maintenance of Hh signal and dorsal-ventral patterning in the neural tube might correspond to a spatiotemporal expression-pattern of *Dyrk2*.

Taken together, *Dyrk2*-deficient embryos exhibit a robust suppression of Hh signaling and possess particular skeletal abnormalities during embryogenesis.

### DYRK2 positively regulates Hh signaling

**Figure 3.**
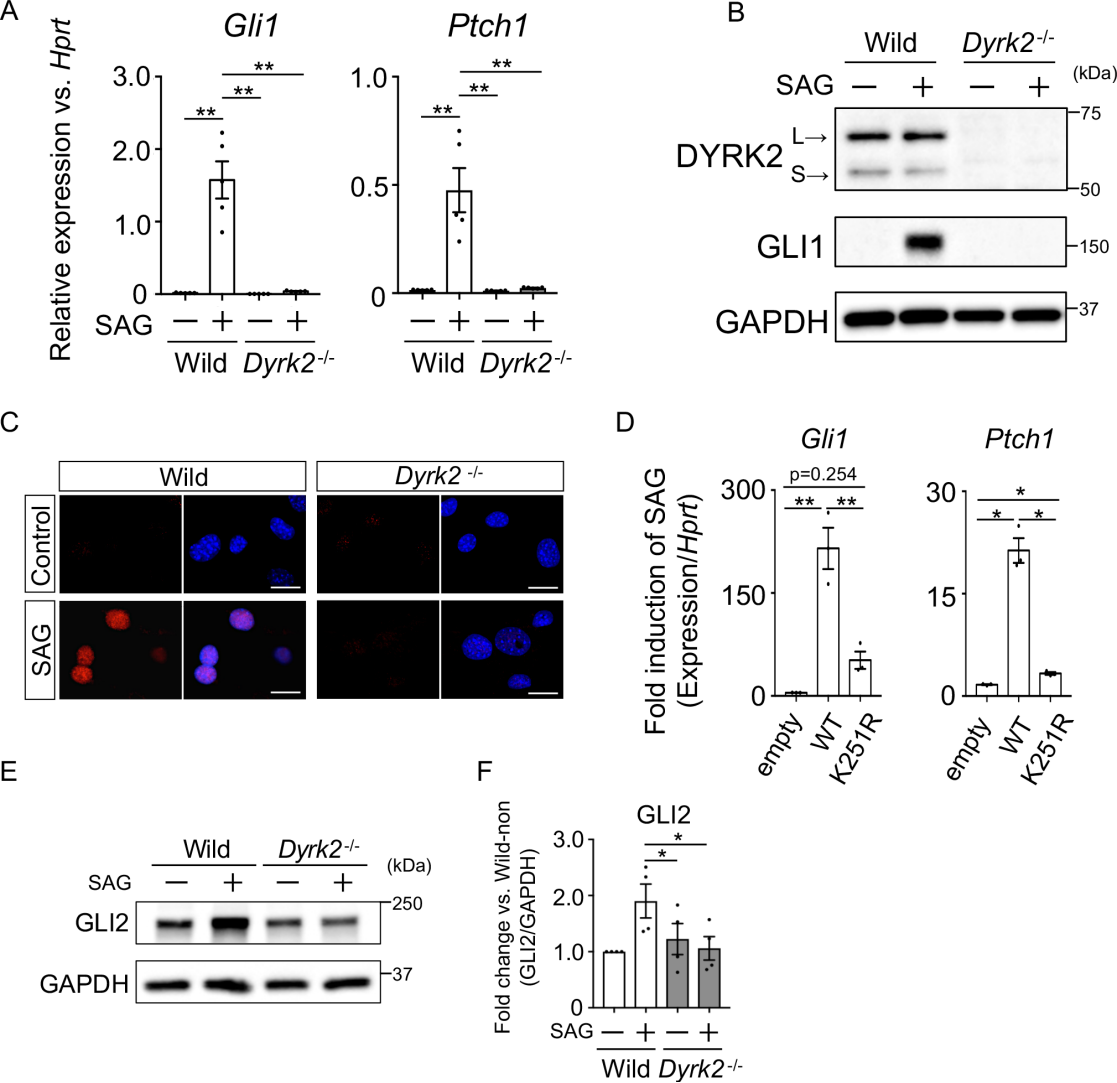
Deletion of *Dyrk2* suppresses activation of Hh signaling in vitro. (**A**) Expression of the Hh target genes *Gli1* and *Ptch1* in wild-type and *Dyrk2^-/-^* MEFs in the absence or presence of 100 nM SAG was measured by qPCR. Data are shown as relative expression to *Hprt*. (**B**) Protein levels of GLI1 and DYRK2 in wild-type and *Dyrk2^-/-^* MEFs in the absence or presence of 100 nM SAG were measured by immuno-blotting. L and S indicate long and short transcriptional isoforms of DYRK2, respectively. (**C**) Wild-type and *Dyrk2^-/-^* MEFs in the absence or presence of 100 nM SAG were immune-cytostained for GLI1 (red). Nuclei were stained with DAPI (blue). Scale bars, 5 µm. (**D**) Expression of *Gli1* and *Ptch1* in *Dyrk2^-/-^* MEFs overexpressing human *DYRK2* or *DYRK2-K251R* (kinase dead) constructs via adenovirus infection was measured by qPCR. Data indicates fold induction of 100 nM SAG against vehicle after normalization to *Hprt*. (**E, F**) Immunoblotting for GLI2 in wild-type and *Dyrk2^-/-^* MEFs in the absence or presence of 100 nM SAG. Protein level as fold changes of GLI2 (**E**) was calculated by comparing protein levels relative to those of wild-type MEFs in the absence of SAG after normalization to the GAPDH loading control in (**F**). Data are presented as the means ± SEM (*n* = 5, 3, and 4 biological replicates per condition in A, D, and F, respectively). The statistical significance was determined by one-way ANOVA followed by Tukey’s multiple comparison test. (*) p<0.05, (**) p<0.01. Figure 3—source data 1.Source data for Figure 3A and D. Figure 3—source data 2.Source data for Figure 3F.

To investigate the defect in Hh signaling in *Dyrk2^-/-^* mice in more detail, we analyzed primary mouse embryonic fibroblasts (MEFs) derived from wild-type and *Dyrk2^-/-^* mice. First, we measured Hh signaling activity in response to stimulation with the SMO agonist SAG. In response to stimulation with SAG, *Gli1* and *Ptch1* expression was increased in wild-type mice as previously reported (Figure 3A; Niewiadomski and Rohatgi, 2015). In contrast, in *Dyrk2^-/-^* MEFs, inductions of both *Gli1* and *Ptch1* expression by SAG was drastically repressed (Figure 3A). Consistent with gene expression analyses, the induction of GLI1 protein by SAG stimulation was also suppressed in *Dyrk2^-/-^* MEFs (Figure 3B). Immunocytostaining for GLI1 following stimulation with SAG demonstrated that the accumulation of GLI1 protein within nuclei was clearly diminished in *Dyrk2^-/-^* MEFs (Figure 3C). To validate whether these phenotypes observed in *Dyrk2^-/-^* MEFs are due to abnormal differentiation caused by deletion of *Dyrk2* during early embryogenesis, we performed a transient knockdown of *Dyrk2* in wild-type MEFs using two independent short interfering RNAs (siRNAs). Transient knockdown of *Dyrk2* also demonstrated suppression of both the mRNA and protein levels of *Gli1* and *Ptch1* in response to stimulation with SAG (Figure 3—figure supplement 1A–B).

**Figure 3—figure supplement 1.**
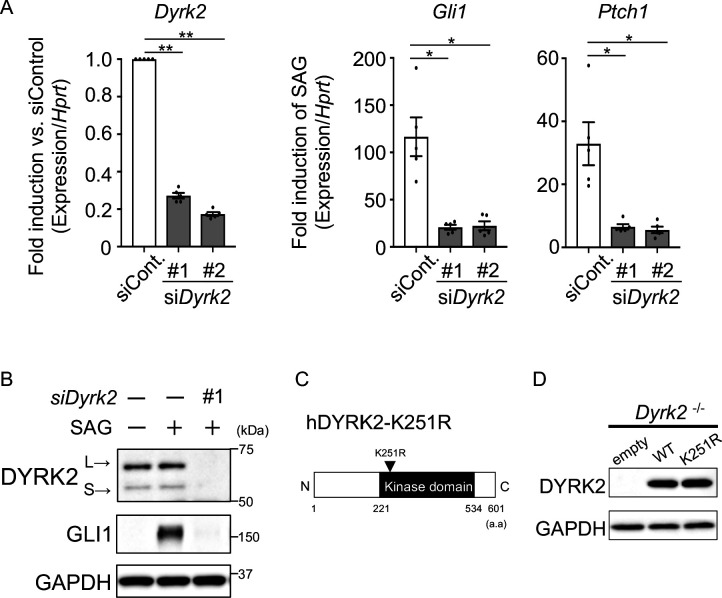
A transient knockdown of *Dyrk2* suppresses activation of Hh signaling. (**A**) Expression of *Gli1* and *Ptch1* in wild-type MEFs treated with two independent si*Dyrk2* for 48 hr was measured by qPCR. *Hprt* was used as an internal standard, and fold change of *Dyrk2* was calculated by comparing expression levels relative to those of siControl. Data for *Gli1* and *Ptch1* indicate fold induction of 100 nM SAG against vehicle after normalization to *Hprt*. Data are presented as the means ± SEM (*n* = 5 biological replicates per condition). The statistical significance was determined by one-way ANOVA followed by Tukey’s multiple comparison test. (*) p<0.05, (**) p<0.01. (**B**) Protein levels of GLI1 and DYRK2 in wild-type MEFs treated with si*Dyrk2* for 48 hr in the absence or presence of 100 nM SAG were measured by immune-blotting. L and S indicate long and short transcriptional isoforms of DYRK2, respectively. (**C**) Schematic representation of a kinase dead human DYRK2 protein. (**D**) Immunoblotting for over-expressed short form of *hDYRK2* or *DYRK2-K251R* (kinase dead) via adenovirus infection in *Dyrk2^-/-^* MEFs. GAPDH serves as a loading control. Figure 3—figure supplement 1—source data 1.Source data for Figure 3—figure supplement 1A.

**Figure 3—figure supplement 2.**
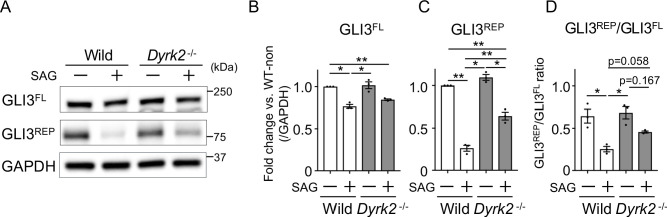
Deletion of *Dyrk2* affects the stabilities of GLI3 Immuno-blotting for GLI3 in wild-type and *Dyrk2^-/-^* MEFs in the absence or presence of 100 nM SAG. Protein levels as fold changes of GLI3^FL^, and GLI3^REP^ (**A**) were calculated by comparing protein levels relative to those of wild-type MEFs in the absence of SAG after normalization to the GAPDH loading control in (**B** and **C**), respectively. The ratio of GLI3^REP^/GLI3^FL^ was calculated directly according to each band intensity value (**D**). Data are presented as the means ± SEM (*n* = 3 biological replicates per condition). The statistical significance was determined by one-way ANOVA followed by Tukey’s multiple comparison test. (*) p<0.05, (**) p<0.01. Figure 3—figure supplement 2—source data 1.Source data for Figure 3—figure supplement 2B–D.

To validate this suppression of Hh signaling by *Dyrk2-*deletion, we performed a transient over-expression experiment using wild-type human *DYRK2* or a *DYRK2-K251R* construct that expresses a kinase dead mutant (Taira et al., 2012; Figure 3—figure supplement 1C–D) in *Dyrk2^-/-^* MEFs using adenovirus infection (Yokoyama-Mashima et al., 2019). Over-expression of the wild-type *DYRK2* construct restored significant induction of *Gli1* and *Ptch1* expression upon exposure to SAG (Figure 3D, Figure 3—figure supplement 1D). In sharp contrast, over-expression of the *DYRK2-K251R* construct in *Dyrk2^-/-^* MEFs markedly diminished *Gli1* and *Ptch1* expression (Figure 3D). Additionally, over-expression of the *DYRK2-K251R* construct slightly increased *Gli1* and *Ptch1* expression in comparison with that of empty vector (Figure 3D). This kinase-independent effect might be associated with a function of DYRK2 as a scaffold protein (Maddika and Chen, 2009). Taken together, the induction of Hh signaling is drastically suppressed by deletion of *Dyrk2* in a kinase activity-dependent manner.

The key Hh pathway components GLI2 and GLI3 are known to be posttranslationally modified in a manner that is dependent upon Hh ligands (Mo et al., 1997; Hui and Angers, 2011). In the absence of Hh ligands, the full-length proteins (active forms; GLI2 and GLI3^FL^) are phosphorylated by multiple kinases, leading to proteasomal degradation or truncation into N-terminal repressor forms (GLI3^REP^), respectively (Niewiadomski and Rohatgi, 2015). In this context, we analyzed the endogenous protein levels and states of GLI2 and GLI3. In wild-type MEFs, immunoblotting for GLI2 revealed that full-length form of GLI2 was increased by SAG-stimulation (Figure 3E–F). In contrast to wild-type MEFs, the increase of GLI2 protein levels by SAG was significantly suppressed in *Dyrk2^-/-^* MEFs (Figure 3E–F). We also analyzed two forms of GLI3 (GLI3^FL^ and GLI3^REP^). SAG-stimulation suppressed the formation of GLI3^REP^ and decreased the ratio of GLI3^REP^/GLI3^FL^ in wild-type MEFs (Figure 3—figure supplement 2; Niewiadomski and Rohatgi, 2015). In *Dyrk2^-/-^* MEFs, however, we found that the ratio of GLI3^REP^/GLI3^FL^ was marginally suppressed and that no significant differences between the absence or presence of SAG existed (Figure 3—figure supplement 2D). Collectively, these data indicate that the deletion of *Dyrk2* affects the stabilities of GLI2, and marginally GLI3, under SAG-stimulation.

### DYRK2 regulates ciliogenesis

**Figure 4.**
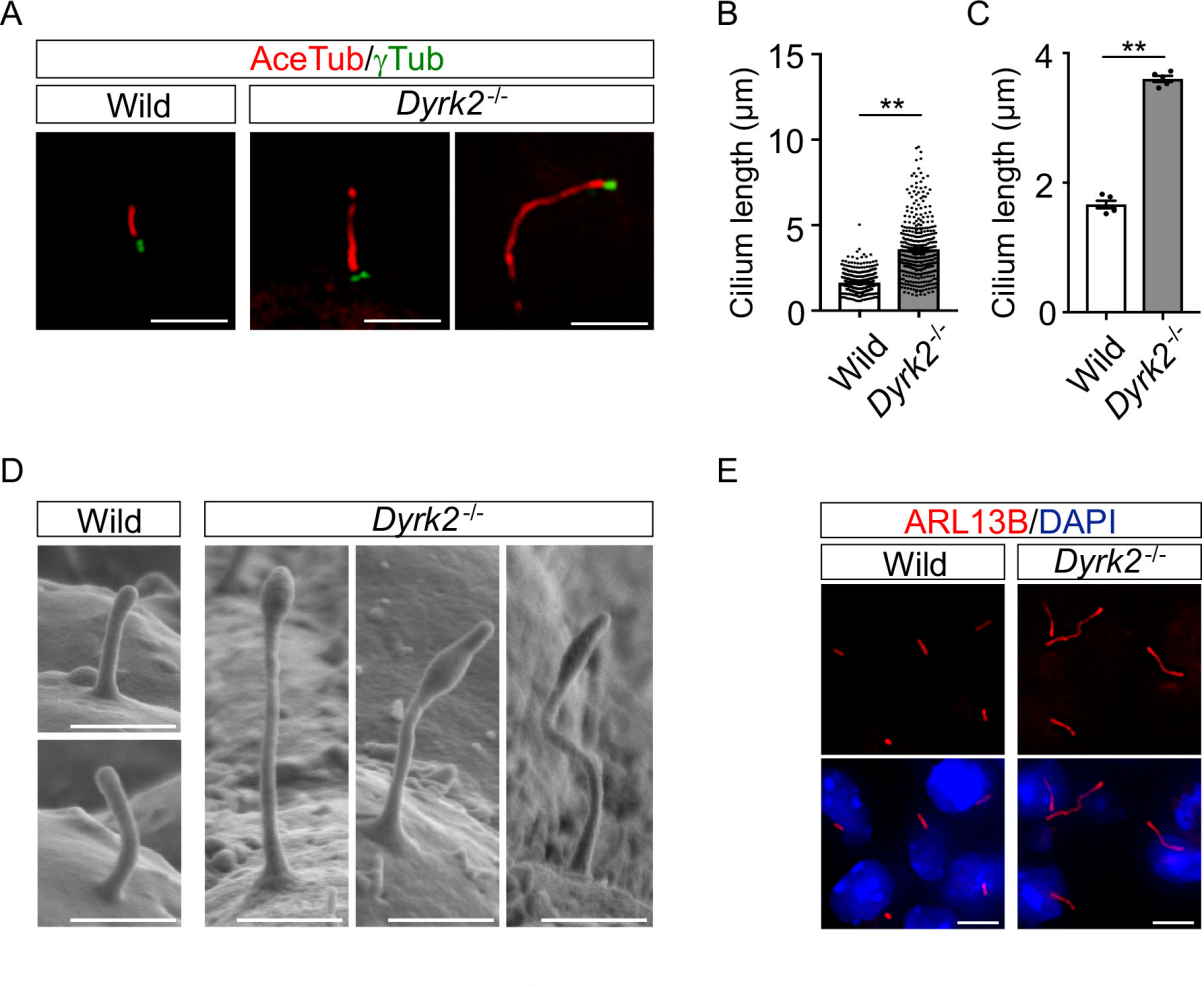
DYRK2 constrains the length of primary cilia. (**A–C**) Elongation of primary cilia in *Dyrk2^-/-^* MEFs. Primary cilia of wild-type and *Dyrk2^-/-^* MEFs were immunostained with acetylated-tubulin and gamma-tubulin antibodies. (**B, C**) Measurements of cilia length in wild-type and *Dyrk2^-/-^* MEFs using acetylated-tubulin as a cilia axoneme marker. Cilia lengths are presented as pooled from five MEFs derived from independent embryos of each genotype (**B**) and the average of each MEF (**C**). Data are presented as the means ± SEM (*n* = 5 biological replicates per condition). The statistical significance between wild-type and *Dyrk2^-/-^* was determined by the Student’s *t*-test. (**) p<0.01. (**D**) Scanning electron microscopy showing wild-type and *Dyrk2^-/-^* embryos in the frontonasal prominence at E10.5. (**E**) Immunohistochemistry of primary cilia in wild-type and *Dyrk2^-/-^* embryos. ARL13B was immuno-stained in wild-type and *Dyrk2^-/-^* mesenchymal cells at the craniofacial region at E13.5. Nuclei were stained with DAPI. Scale bars, 5 µm (**A and E**) and 1 µm (**D**). Figure 4—source data 1.Source data for Figure 4B–C.

As primary cilia are essential organelles required for signal transduction of vertebrate Hh signaling (Huangfu et al., 2003), we investigated whether DYRK2 regulates ciliogenesis in MEFs. Immunostaining of acetylated tubulin (a cilia axoneme marker) and γ-tubulin (a basal body marker) demonstrated that the length of primary cilia in *Dyrk2^-/-^* MEFs was significantly longer than that in wild-type MEFs (Figure 4A). The average cilia length in wild-type MEFs was 1.65 ± 0.03, while it was 3.59 ± 0.08 in *Dyrk2^-/-^* MEFs (Figure 4B–C). In addition to the increased length, the morphology of primary cilia in *Dyrk2^-/-^* MEFs was often bulged at the tips, tapered, and twisted (Figure 4A). This elongation and morphological abnormality of primary cilia was similarly observed in response to the transient knockdown of *Dyrk2* by siRNA in wild-type MEFs (Figure 4—figure supplement 1). To investigate whether the regulation of ciliogenesis by DYRK2 is conserved in other species and cell types, we analyzed immortalized human retinal pigment epithelia cells (hTERT-RPE1 cells) that are commonly used to study cilia-assembly and -disassembly. A transient knockdown by si*DYRK2* also induced a significant elongation and morphological abnormality in cilia of these cells (Figure 4—figure supplement 2). In contrast to the length and morphology of primary cilia, no difference was observed on the proportion of ciliated cells in wild-type and *Dyrk2^-/-^* MEFs (Figure 4—figure supplement 3A–B). Similarly, in cell-cycling (KI67-positive) wild-type and *Dyrk2^-/-^* MEFs, there was comparable in the proportion of ciliated cells (ciliated cells in KI67-positive cells is 1 per 199 and 1 per 139 cells in wild-type and *Dyrk2^-/-^* MEFs, respectively) (Figure 4—figure supplement 3C).

**Figure 4—figure supplement 1.**
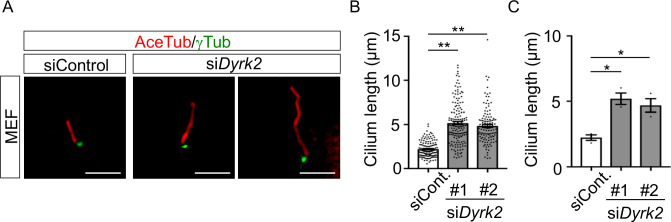
Elongation of primary cilia in wild-type MEFs treated with si*Dyrk2.* (**A**) Primary cilia of wild-type and *Dyrk2^-/-^* MEFs treated with siControl or two independent si*Dyrk2* were immunostained with acetylated-tubulin and gamma-tubulin antibodies. Scale bars, 5 µm. (**B, C**) Measurements of cilia length in wild-type MEFs treated with siControl or si*Dyrk2* using acetylated-tubulin as a cilia axoneme marker. Cilia lengths are presented as pooled from three MEFs derived from independent wild-type embryos (**B**) and represent the average of each MEF (**C**). Data are presented as the means ± SEM (*n* = 3 biological replicates per condition). The statistical significance was determined by one-way ANOVA followed by Tukey’s multiple comparison test. (*) p<0.05, (**) p<0.01. Figure 4—figure supplement 1—source data 1.Source data for Figure 4—figure supplement 1B–C.

**Figure 4—figure supplement 2.**
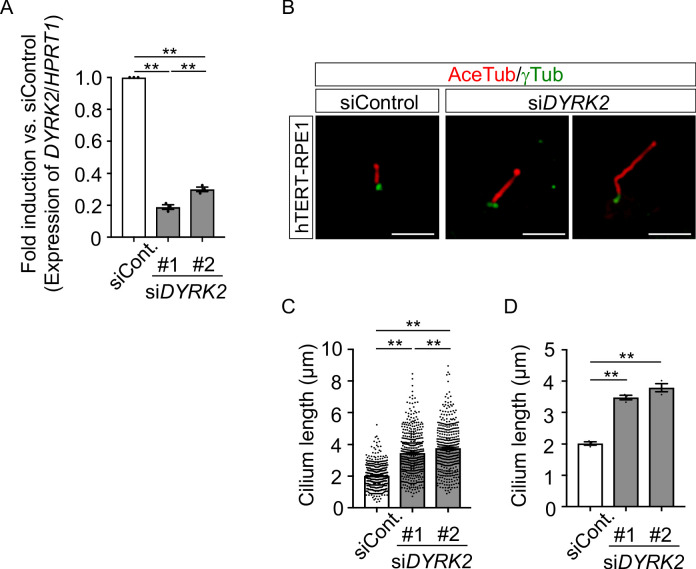
Elongation of primary cilia in hTERT-RPE1 cells treated with si*DYRK2*. (**A**) Knockdown efficiency of *DYRK2*-expression in hTERT-RPE1 cells treated with two independent si*DYRK2* for 48 hr was measured by qPCR. *HPRT1* was used as an internal standard, and fold change was calculated by comparing expression levels relative to those of siControl. (**B**) Primary cilia of hTERT-RPE1 cells treated with siControl or si*DYRK2* were immunostained with acetylated-tubulin and gamma-tubulin antibodies. (**C, D**) Measurements of cilia length in hTERT-RPE1 cells treated with siControl or two independent si*DYRK2* using acetylated-tubulin as a cilia axoneme marker. Scale bars, 5 µm. Cilia lengths are presented as pooled from three independent experiments (**C**) and represent the average of each condition (**D**). Data are presented as the means ± SEM (*n* = 3 replicates per condition). The statistical significance was determined by one-way ANOVA followed by Tukey’s multiple comparison test. (**) p<0.01. Figure 4—figure supplement 2—source data 1.Source data for Figure 4—figure supplement 2A and C–D.

**Figure 4—figure supplement 3.**
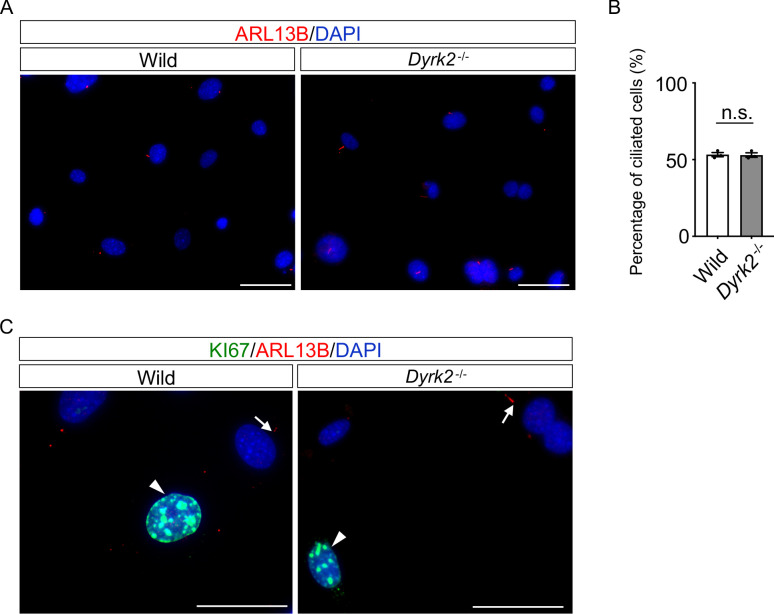
Quantification of the proportion of ciliated cells in wild-type and *Dyrk2^-/-^* MEFs. (**A, B**) Proportion of ciliated cells in wild-type and *Dyrk2^-/-^* MEFs. Primary cilia of wild-type and *Dyrk2^-/-^* MEFs were immunostained with ARL13B (**A**). Measurements of proportion of ciliated cells in wild-type and *Dyrk2^-/-^* MEFs using ARL13B as a cilia axoneme marker (**B**). Data are presented as the means ± SEM (*n* = 3 biological replicates per condition;>150 cells were scored for each experiment). The statistical significance between wild-type and *Dyrk2^-/-^* was determined by the Student’s *t*-test. (**C**) Proportion of ciliated cells in cell-cycling wild-type and *Dyrk2^-/-^* MEFs. Wild-type and *Dyrk2^-/-^* MEFs cultured under 10% FBS containing medium at low density were immunostained with KI67 and ARL13B. Nuclei were stained with DAPI. Arrowheads and arrows indicate non-ciliated/KI67-positive cycling cells and ciliated/KI67-negative ones, respectively. Scale bars, 50 µm. Figure 4—figure supplement 3—source data 1.Source data for Figure 4—figure supplement 3B.

To confirm the morphological abnormalities of primary cilia within the tissue, we performed scanning electron microscopy (SEM) on embryos at E10.5. SEM images clearly showed that the cilia of *Dyrk2^-/-^* embryos were significantly elongated, bulged at the tips, and twisted, while those of wild-type embryos were shortened and straight (Figure 4D). These abnormalities were also observed in several types of cells, including mesenchymal cells (Figure 4E), chondrocytes, neuroepithelium, and tongue cells (data not shown) in the embryonic craniofacial region at E13.5 as assessed by immunohistochemistry.

**Figure 5.**
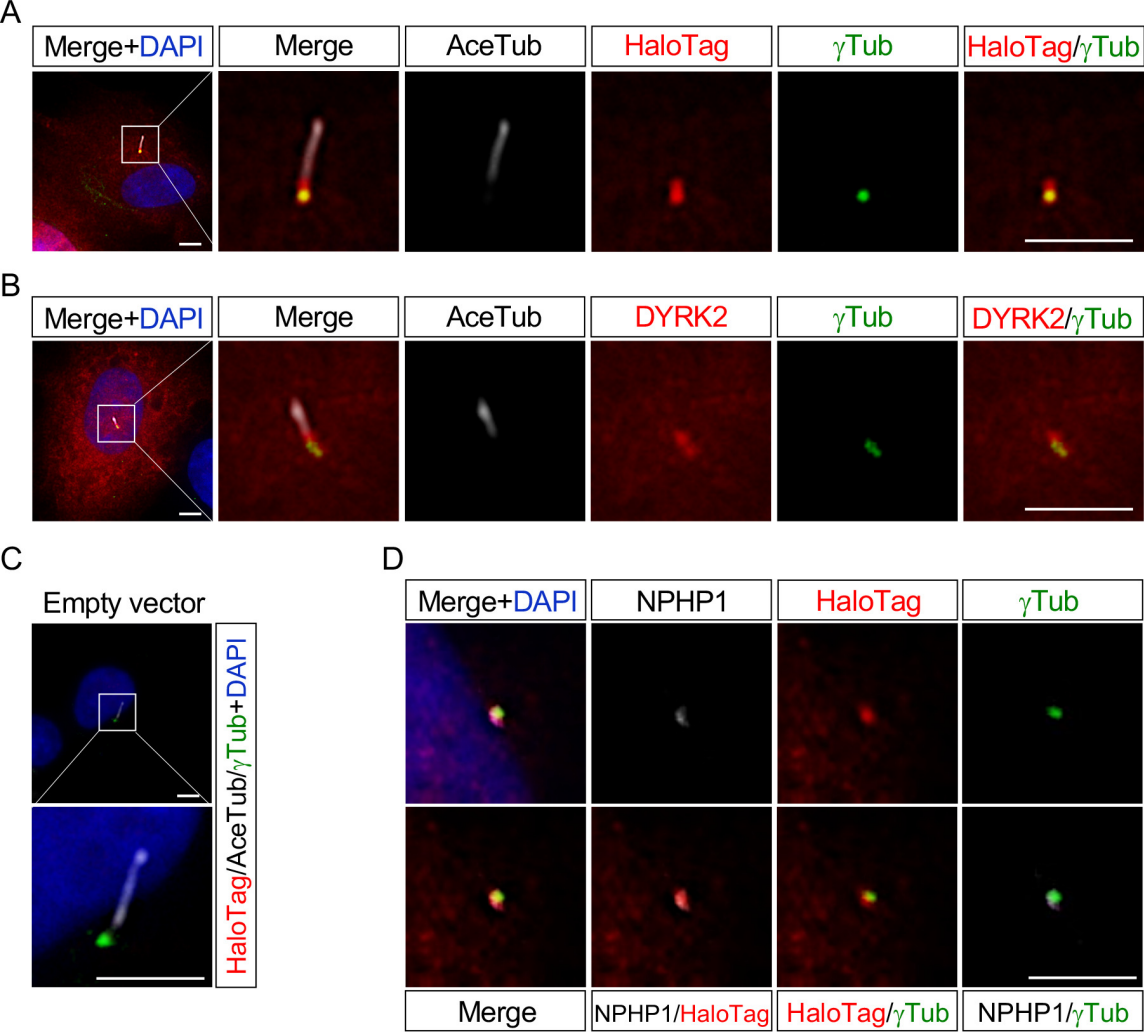
DYRK2 localizes at basal bodies and transition zone (TZ) in primary cilia. Cultured hTERT-RPE1 cells were transfected with a mouse DYRK2-HaloTag overexpression construct and immunostained using anti-HaloTag (**A**) or anti-DYRK2 (**B**) with acetylated-tubulin (white) and gamma-tubulin antibodies. (**C**) Cultured hTERT-RPE1 cells transfected with an empty vector (pFN22K-Halo Tag-CMVd1-Flexi-vector) and immunostained using anti-HaloTag with acetylated-tubulin (white) and gamma-tubulin antibodies. (**D**) Co-localization of DYRK2 and a TZ marker, NPHP1. Cultured hTERT-RPE1 cells overexpressed with a mouse DYRK2-HaloTag were immunostained using anti-HaloTag, NPHP1 (white), and gamma-tubulin antibodies. Nuclei were stained with DAPI. Scale bars, 5 µm.

These data indicating that deletion of *Dyrk2* causes morphological abnormalities in primary cilia prompted us to determine the subcellular localization of DYRK2. We transfected DYRK2-HaloTag constructs into hTERT-RPE1 cells and induced ciliogenesis by serum-starvation. Immunocytostaining for both anti-HaloTag and anti-DYRK2 revealed that DYRK2 localized at γ-tubulin-positive basal bodies and at the proximal end of the axoneme, namely a transition zone (TZ) (Figure 5A–B). No signal for anti-HaloTag (Figure 5C) or anti-DYRK2 (data not shown) was observed in hTERT-RPE1 cells transfected with empty vector (pFN22K-Halo Tag-CMVd1-Flexi-vector). Moreover, immuno-positive signals for DYRK2-HaloTag were co-localized with a TZ marker, NPHP1 (Figure 5D).

These data indicated that DYRK2 might regulate ciliogenesis at basal bodies and TZ in vivo and in vitro.

### Deletion of DYRK2 induces abnormal ciliary trafficking of Hedgehog pathway components

**Figure 6.**
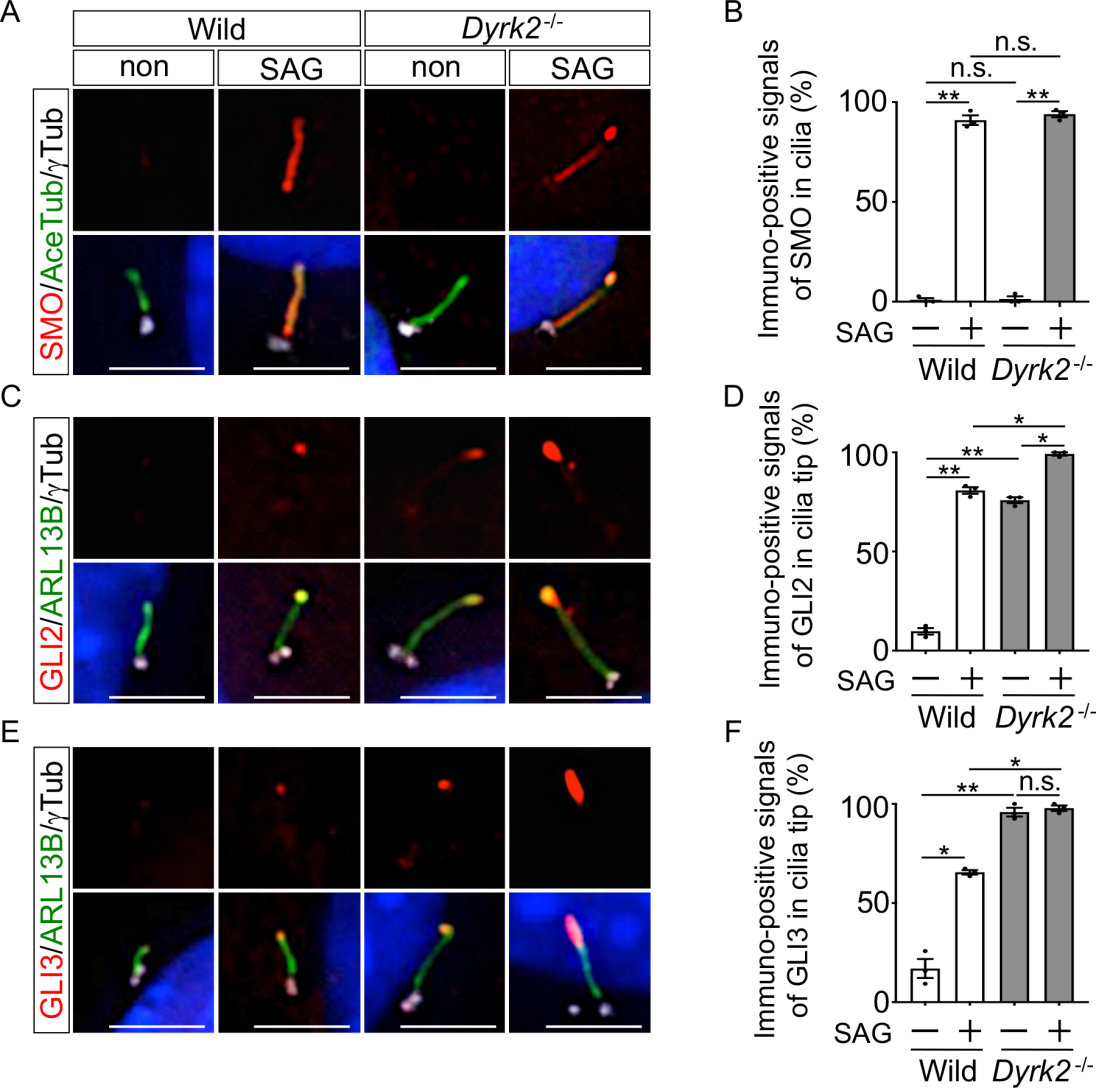
Depletion of *Dyrk2* induces abnormal ciliary trafficking of endogenous Hh components. Ciliary localization of endogenous SMO, GLI2, and GLI3 in wild-type and *Dyrk2^-/-^* MEFs in the absence or presence of 100 nM SAG. Primary cilia were immuno-stained for SMO (**A**), GLI2 (**C**), or GLI3 (**E**) with ARL13B and gamma-tubulin (white) antibodies. Nuclei were stained with DAPI (blue). The percentage of cells with SMO (**B**) at the cilia or foci of GLI2 (**D**) or GLI3 (**F**) at the cilia tips was determined. Data are presented as the means ± SEM (*n* = 3 biological replicates for each condition;>110 cells were scored for each experiment). The statistical significance was determined by one-way ANOVA followed by Tukey’s multiple comparison test. (*) p<0.05, (**) p<0.01. Scale bars, 5 µm. Figure 6—source data 1.Source data for Figure 6B,D and F.

In mammals, key regulators of Hh signaling have been demonstrated to be recruited and activated at the cilia upon Hh stimulation (Chen et al., 2009; Kim et al., 2009; Tukachinsky et al., 2010; Wen et al., 2010), and disorders in the ciliary trafficking of Hh components cause dysfunction of Hh signaling (He et al., 2014). To investigate whether inactivation of Hh signaling in *Dyrk2^-/-^* embryos and MEFs is due to abnormal ciliary trafficking of Hh components, we analyzed the ciliary localization of key regulators such as SMO, GLI2, GLI3, and SuFu. In wild-type MEFs, immuno-positive SMO signals were mostly undetectable or faint in the absence of Hh stimulation, and recruitment to cilia was dependent upon Hh stimulation (Figure 6A–B), as shown in a previous report (Tukachinsky et al., 2010). Similarly, no significant difference in the frequency of SMO recruitment in response to SAG stimulation was observed in *Dyrk2^-/-^* MEFs (Figure 6A–B).

**Figure 6—figure supplement 1.**
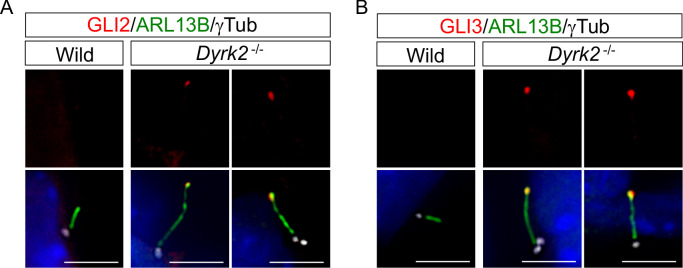
Depletion of *Dyrk2* induces abnormal ciliary trafficking of endogenous GLI2 and GLI3 in vivo. Immunohistochemistry for GLI2 and GLI3 in wild-type and *Dyrk2^-/-^* mesenchymal cells in the craniofacial region at E10.5 tissues. Primary cilia were immuno-stained for GLI2 (**A**) or GLI3 (**B**) with ARL13B and gamma-tubulin (white) antibodies. Nuclei were stained with DAPI (blue). Scale bars, 5 µm.

**Figure 6—figure supplement 2.**
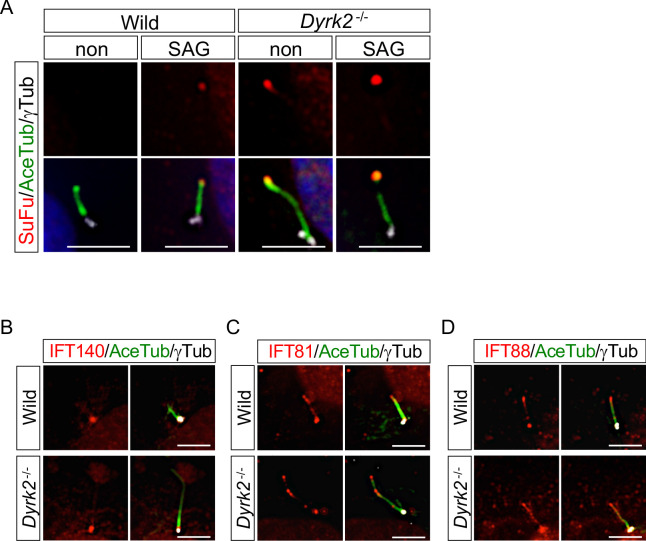
Immunocytochemistry of endogenous SuFu and IFTs. (**A**) Ciliary localization of endogenous SuFu in wild-type and *Dyrk2^-/-^* MEFs in the absence or presence of 100 nM SAG. Primary cilia were immunostained for SuFu with ARL13B and gamma-tubulin (white) antibodies. (**B–D**) Ciliary localization of endogenous IFTs in wild-type and *Dyrk2^-/-^* MEFs. Primary cilia were immuno-stained for IFT140 (**B**), IFT81 (**C**), or IFT88 (**D**) with acetylated-tubulin and gamma-tubulin (white) antibodies. Nuclei were stained with DAPI (blue). Scale bars, 5 µm.

**Figure 6—figure supplement 3.**
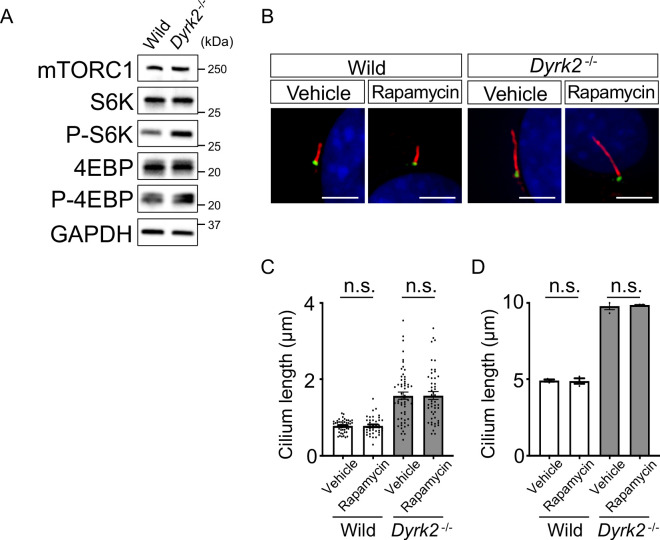
Effects of rapamycin treatment on cilia. (**A**) Phosphorylated protein levels of S6K and 4EBP in wild-type and *Dyrk2^-/-^* MEFs were measured by immunoblotting. GAPDH serves as a loading control. (**B**) Primary cilia in wild-type and *Dyrk2^-/-^* MEFs treated with vehicle (DMSO) or 0.5 µM rapamycin for 24 hr were immunostained with acetylated-tubulin (red) and gamma-tubulin (green) antibodies. Nuclei were stained with DAPI (blue). Scale bars, 5 µm. (**C, D**) Measurements of cilia length in wild-type and *Dyrk2^-/-^* MEFs treated with vehicle (DMSO) or 0.5 µM rapamycin using acetylated-tubulin as a cilia axoneme marker. Cilia lengths are presented as pooled from three MEFs derived from independent embryos of each genotype (**C**) and the average of each MEF (**D**). Data are presented as the means ± SEM (*n* = 3 biological replicates per condition). The statistical significance was determined by one-way ANOVA followed by Tukey’s multiple comparison test. Figure 6—figure supplement 3—source data 1.Source data for Figure 6—figure supplement 3C–D.

**Figure 6—figure supplement 4.**
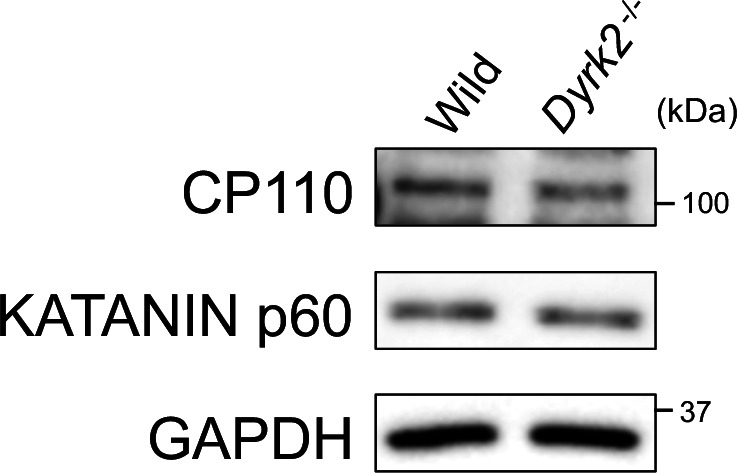
Protein levels of CP110 and KATANIN p60 in *Dyrk2^-/-^* MEFs. Protein levels of CP110 and KATANIN p60 in wild-type and *Dyrk2^-/-^* were measured by immune-blotting. GAPDH serves as a loading control.

We subsequently analyzed the ciliary localization of endogenous GLI2 and GLI3. As shown in a previous report (Tukachinsky et al., 2010), in wild-type MEFs, immuno-positive signals for both GLI2 and GLI3 were mostly undetectable or faint in the absence of Hh stimulation, and SAG stimulation increased the presence of GLI2 and GLI3 at cilia tips (Figure 6C–F). In contrast, in *Dyrk2^-/-^* MEFs, immuno-positive signals for both GLI2 (approximately 75.9% of cilia) and GLI3 (approximately 95.8%) were observed at cilia tip even in the absence of SAG treatment (Figure 6C–F). Moreover, the intensity of immune-positive signals was markedly increased by SAG-treatment in *Dyrk2^-/-^* MEFs (Figure 6C,E). These accumulations at cilia tips were frequently observed in *Dyrk2^-/-^* mesenchymal cells in the craniofacial region at E10.5 tissues but were absent in wild-type cells (Figure 6—figure supplement 1). Additionally, localization of SuFu, which forms a complex with both GLI2 and GLI3 (Tukachinsky et al., 2010), was disordered in cilia tips in a similar pattern to that of GLI2 and GLI3 (Figure 6—figure supplement 2A). Collectively, ciliary localization of GLI2, GLI3, and SuFu in *Dyrk2^-/-^* MEFs and embryos was clearly disordered and was accumulated at cilia tips. Importantly, the recruitment of GLI2, GLI3, and SuFu in response to SAG stimulation was also observed in *Dyrk2^-/-^* MEFs.

Inhibition of retrograde transport from the tip to the cell body induces accumulation of Hh components and results in abnormal localization of both IFT-A (implicated in retrograde IFT) and IFT-B (implicated in anterograde) (Ocbina and Anderson, 2008; Ocbina et al., 2011; Liem et al., 2012). Based on this, we analyzed the ciliary localization of core IFT-A (IFT140) and IFT-B (IFT81 and IFT88) (Nakayama and Katoh, 2018). In *Dyrk2^-/-^* MEFs, no obvious differences in ciliary localization of IFT140, IFT81, and IFT88 were observed (Figure 6—figure supplement 2B–D).

Activation of mammalian target of rapamycin complex 1 (mTORC1) also induces abnormal trafficking and elongates cilia length (Broekhuis et al., 2014). mTORC1 activation leads to phosphorylation of ribosomal S6 kinase (S6K) and eukaryotic translational initiation factor 4E binding protein (4EBP). In *Dyrk2^-/-^* MEFs, phosphorylation of both S6K and 4EBP was slightly increased (Figure 6—figure supplement 3A); however, treatment with rapamycin, an inhibitor of mTORC1, resulted in no obvious differences in cilia length in *Dyrk2^-/-^* MEFs (Figure 6—figure supplement 3B–D).

Moreover, a centrosome protein CP110 (Hossain et al., 2017) and a microtubule severing enzyme, KATANIN p60 (Maddika and Chen, 2009), have been identified as substrates of DYRK2 for proteolysis. In *Dyrk2^-/-^* MEFs, however, no obvious difference in protein levels of both CP110 and KATANIN p60 was observed (Figure 6—figure supplement 4).

### Deletion of *Dyrk2* dysregulates the expression of *Aurka* and other cilia-disassembly genes

**Figure 7.**
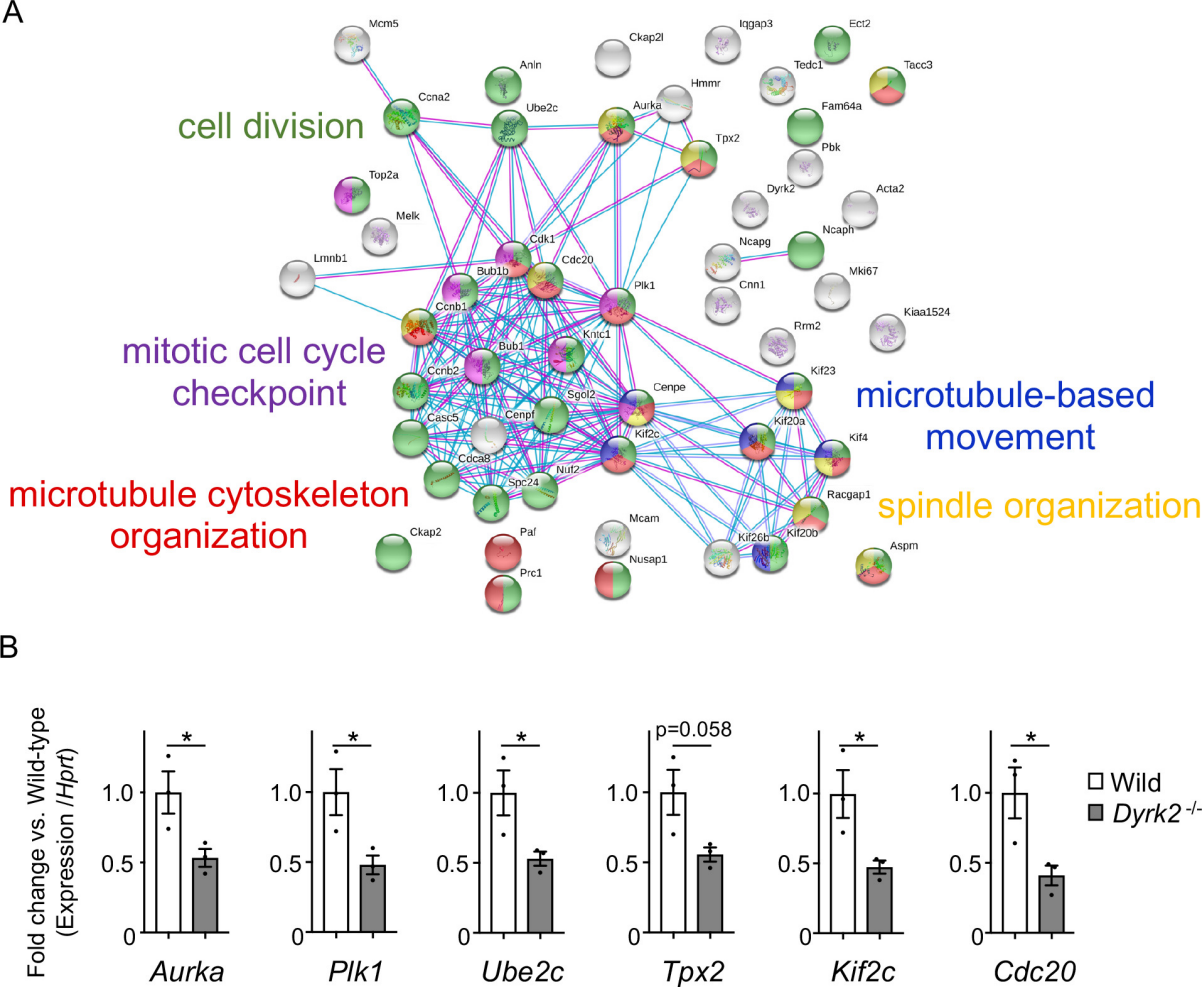
Changes in mRNA expression of genes in *Dyrk2^-/-^* MEFs. (**A**) STRING GO analyses of the 53 differentially downregulated genes in *Dyrk2^-/-^* MEFs reveals protein-protein interaction networks. Robust networks for cell division (green, GO: 0051301), microtubule cytoskeleton organization (red, GO:0000226), spindle organization (yellow, GO:0007051), mitotic cell cycle checkpoint function (purple, GO:0007093), and microtubule-based movement (blue, GO:0007018) were extracted. (**B**) Confirmation of downregulation of genes related to ciliary resorption mechanisms in *Dyrk2^-/-^* MEFs by qPCR. *Hprt* was used as an internal standard, and fold change was calculated by comparing expression levels relative to those of wild-type. Data are presented as the means ± SEM (*n* = 3 biological replicates per condition). The statistical significance between wild-type and *Dyrk2^-/-^* MEFs was determined using the Student’s *t*-test. (*) p<0.05. Figure 7—source data 1.Source data for Figure 7B.

**Figure 8.**
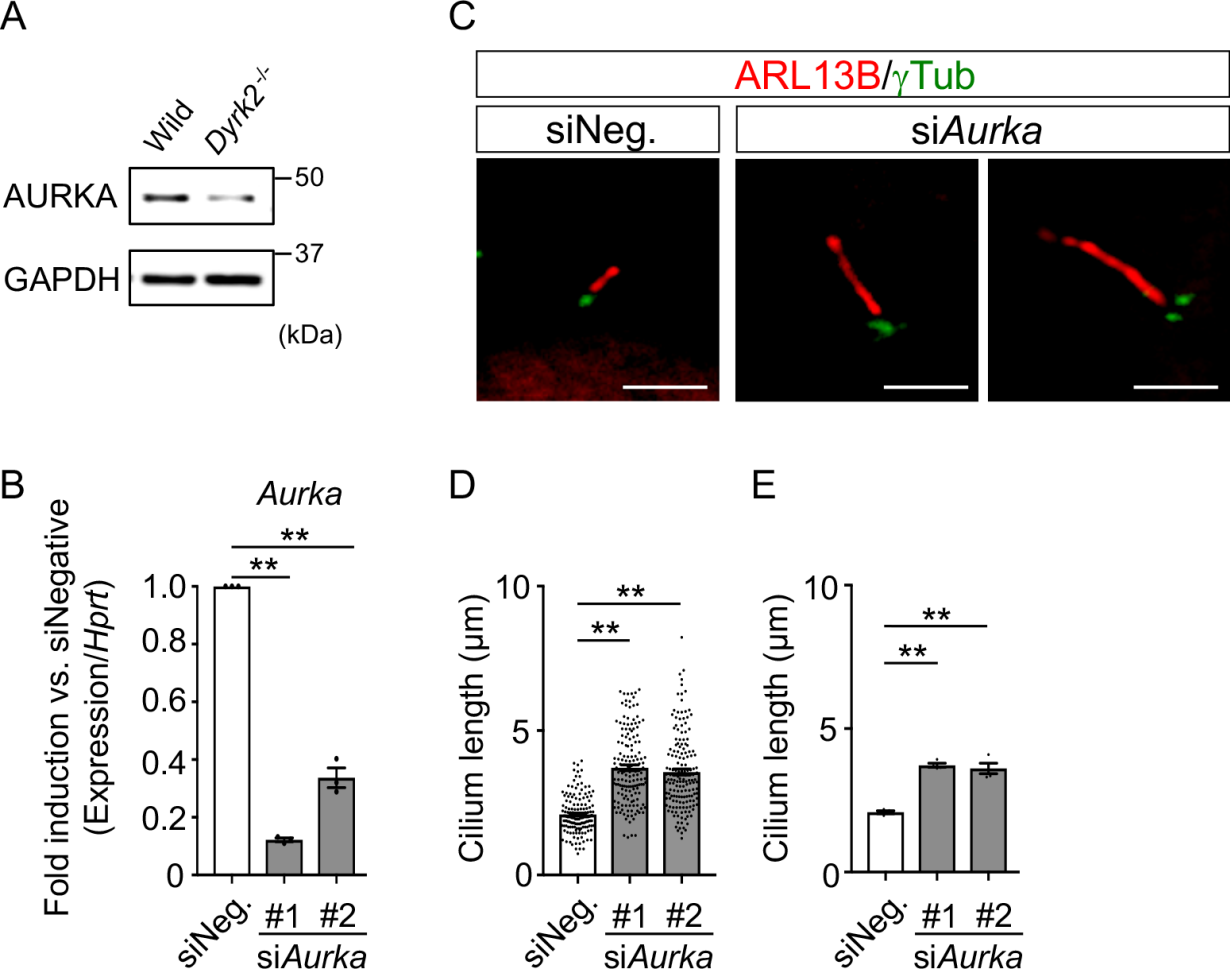
Elongation of primary cilia in wild-type MEFs treated with si*Aurka.* (**A**) Immunoblotting of AURKA in wild-type and *Dyrk2^-/-^* MEFs. GAPDH serves as a loading control. (**B**) Knockdown efficiency of *Aurka*-expression in wild-type MEFs treated with two independent si*Aurka* for 48 hr was measured by qPCR. *Hprt* was used as an internal standard, and fold change was calculated by comparing expression levels relative to those of siNegative (siNeg.). Data are presented as the means ± SEM (*n* = 3 biological replicates per condition). (**C**) Primary cilia in wild-type cells treated with siNegative (siNeg.) or two independent si*Aurka* were immuno-stained with ARL13B and gamma-tubulin antibodies. Scale bars, 5 µm. (**D, E**) Measurements of cilia length in wild-type MEFs treated with siNeg. or two independent si*Aurka* using ARL13B and acetylated-tubulin as a cilia axoneme marker. Cilia lengths are presented as pooled from four MEFs derived from independent wild-type embryos (**D**) and represent an average of each MEF (**E**). Data are presented as the means ± SEM (*n* = 4 biological replicates per condition). The statistical significance was determined by one-way ANOVA followed by Tukey’s multiple comparison test. (**) p<0.01. Figure 8—source data 1.Source data for Figure 8B and D–E.

**Figure 9.**
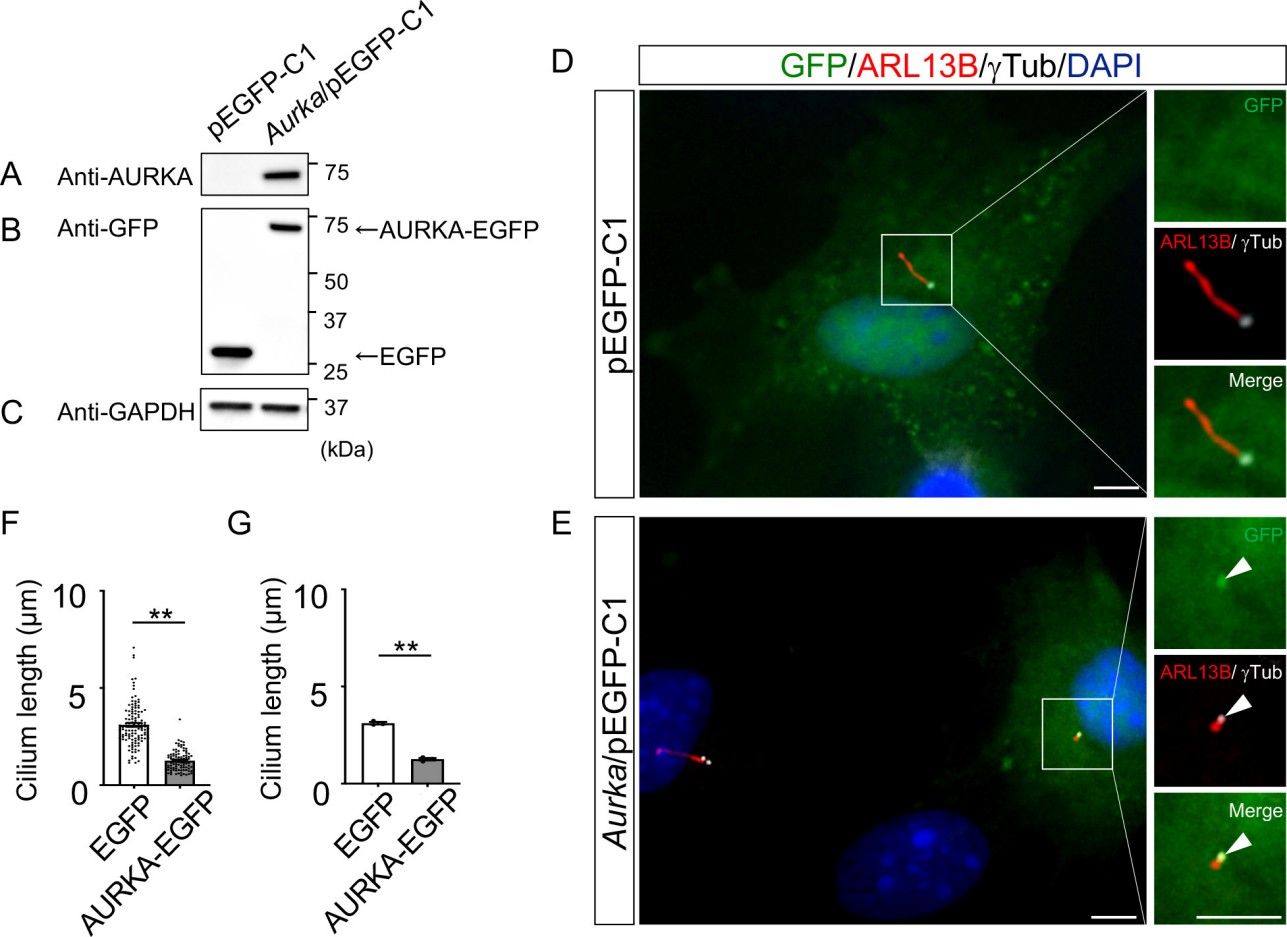
Reduction of the length of primary cilia in *Dyrk2^-/-^* MEFs by over-expression of AURKA. (**A–C**) Immunoblotting by anti-AURKA (**A**), anti-GFP (**B**), and anti-GAPDH (**C**) in cells transfected with pEGFP-C1 or mouse *Aurka*/pEGFP-C1. GAPDH serves as a loading control. (**D, E**) Primary cilia in *Dyrk2^-/-^* MEFs over-expressed with EGFP (**D**) or AURKA-EGFP (**E**) were immunostained with GFP, ARL13B, and gamma-tubulin (white) antibodies. Arrowheads in (**E**) indicate signals for AURKA-EGFP in gamma-tubulin-positive basal body. Scale bars, 5 µm. (**F, G**) Measurements of cilia length in EGFP- or AURKA-EGFP-over-expressed *Dyrk2^-/-^* MEFs using ARL13B as a cilia axoneme marker. Cilia lengths in EGFP- or AURKA-EGFP-positive cells are presented as pooled from three MEFs derived from independent *Dyrk2^-/-^* embryos (**F**) and represent an average of each MEF (**G**). Data are presented as the means ± SEM (*n* = 3 biological replicates per condition). The statistical significance between EGFP- and AURKA-EGFP-positive cells was determined by the Student’s *t*-test. (**) p<0.01. Figure 9—source data 1.Source data for Figure 9F and G.

To understand the molecular mechanisms underlying cilia dysfunction in *Dyrk2^-/-^* mice, we focused on factors that are involved in ciliary length control by incorporating whole-genome RNA sequencing using wild-type and *Dyrk2^-/-^* MEFs (Figure 7, Figure 7—figure supplement 1). The data were analyzed by multiple testing and according to p-value, false discovery rate (FDR), and ratio (*Dyrk2^-/-^*/wild-type). As a result, the number of identified genes was 53 or 42 that were significantly downregulated (p<0.005, ratio <1.5 fold) or upregulated (p<0.005, ratio >1.5 fold) in *Dyrk2^-/-^* MEFs, respectively, regardless of the presence or absence of SAG (Table 1). Notably, GO and STRING analysis revealed that the 53 downregulated genes in *Dyrk2^-/-^* MEFs were enriched in cell division (GO: 0051301, FDR = 5.17E-40), microtubule cytoskeleton organization (GO:0000226, FDR = 5.23E-15), spindle organization (GO:0007051, FDR = 2.72E-11), mitotic cell cycle checkpoint (GO:0007093, FDR = 3.78E-07), and microtubule-based movement (GO:0007018, FDR = 3.9E-4) (Figure 7A, Figure 7—figure supplement 1B). These downregulated genes in *Dyrk2^-/-^* MEFs included those related to ciliary resorption mechanisms for proliferation, including the HEF1-AURKA-HDAC6 pathway (*Aurka*, *Plk1*, *Ube2c*, and *Tpx2*) (Pugacheva et al., 2007), the PLK-KIF2A pathway (*Plk1* and *Kif2c*, a family of *Kif2a*) (Wang et al., 2013), and the APC^CDC20^-Nek1 pathway (*Cdc20*) that controls ciliary length (Wang et al., 2014; Keeling et al., 2016). We confirmed the downregulation of *Aurka*, *Plk1*, *Ube2c*, *Tpx2, Kif2c,* and *Cdc20* in *Dyrk2^-/-^* MEFs by qPCR (Figure 7B). To identify a molecule involved in cilia-elongation in *Dyrk2^-/-^* cells, we performed transient knockdown of selected genes using siRNA in wild-type MEFs, and we analyzed cilia length. Notably, we found that knockdown of *Aurka* by two independent siRNAs significantly increases cilia length (Figure 8). Moreover, we performed a rescue experiment by over-expression of AURKA-EGFP in *Dyrk2^-/-^* MEFs (Figure 9). Immunostaining and measurement of the cilia length in EGFP- (transfected with pEGFP-C1) or AURKA-EGFP-positive (transfected with *Aurka*/pEGFP-C1) *Dyrk2^-/-^* MEFs demonstrated that elongated cilia in *Dyrk2^-/-^* MEFs were significantly shortened in AURKA-EGFP-positive cells in comparison with EGFP-positive ones (Figure 9D–G).

**Figure 7—figure supplement 1.**
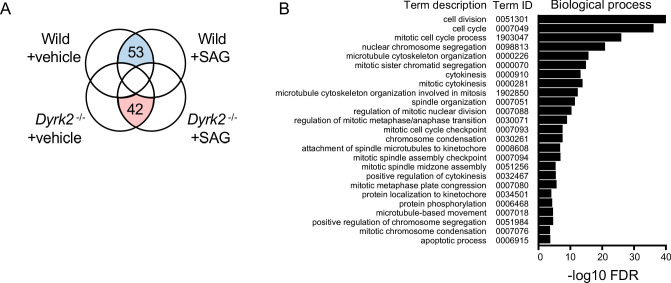
Transcriptome analysis in *Dyrk2^-/-^* MEFs. (**A**) Venn diagrams revealing the similarities and differences among genes that were differentially expressed more than 1.5-fold from RNA-seq experiments in wild-type and *Dyrk2^-/-^* MEFs in the absence or presence of 100 nM SAG. (**B**) Significantly enriched gene ontology terms belonging to ‘biological process’ (false discovery rate: FDR < 0.005) among 53 differentially downregulated genes in *Dyrk2^-/-^* MEFs.

**Table 1.** A list of downregulated or upregulated genes in Dyrk2^-/-^ MEFs

Down-regulated genes in Dyrk2^-/-^				
ID	GeneSymbol	Description	Ratio of Dyrk2^-/-^per wild-type in the presence of SAG	Ratio of Dyrk2^-/-^per wild-type in the absence of SAG
ENSMUSG00000028630	Dyrk2	Dual-specificity tyrosine-(Y)-phosphorylation regulated kinase 2	0.02	0.03
ENSMUSG00000035683	Melk	Maternal embryonic leucine zipper kinase	0.23	0.22
ENSMUSG00000074476	Spc24	NDC80 kinetochore complex component%2C homolog (*S. cerevisiae*)	0.25	0.21
ENSMUSG00000020808	Pimreg	PICALM interacting mitotic regulator	0.28	0.28
ENSMUSG00000033952	Aspm	Abnormal spindle microtubule assembly	0.31	0.25
ENSMUSG00000026683	Nuf2	NDC80 kinetochore complex component	0.31	0.30
ENSMUSG00000037466	Tedc1	Tubulin epsilon and delta complex 1	0.31	0.26
ENSMUSG00000030867	Plk1	Polo-like kinase 1	0.31	0.17
ENSMUSG00000022033	Pbk	PDZ binding kinase	0.33	0.29
ENSMUSG00000027326	Knl1	Kinetochore scaffold 1	0.33	0.20
ENSMUSG00000041431	Ccnb1	Cyclin B1	0.33	0.26
ENSMUSG00000036777	Anln	Anillin actin binding protein	0.33	0.26
ENSMUSG00000001403	Ube2c	Ubiquitin-conjugating enzyme E2C	0.33	0.25
ENSMUSG00000027496	Aurka	Aurora kinase A	0.34	0.26
ENSMUSG00000001349	Cnn1	Calponin 1	0.34	0.31
ENSMUSG00000032218	Ccnb2	Cyclin B2	0.34	0.28
ENSMUSG00000026039	Sgo2a	Shugoshin 2A	0.34	0.25
ENSMUSG00000015880	Ncapg	Non-SMC condensin I complex subunit G	0.34	0.34
ENSMUSG00000027379	Bub1	BUB1 mitotic checkpoint serine/threonine kinase	0.36	0.23
ENSMUSG00000040084	Bub1b	BUB1B mitotic checkpoint serine/threonine kinase	0.36	0.29
ENSMUSG00000045328	Cenpe	Centromere protein E	0.36	0.22
ENSMUSG00000032254	Kif23	Kinesin family member 23	0.37	0.25
ENSMUSG00000028873	Cdca8	Cell division cycle associated 8	0.37	0.30
ENSMUSG00000032135	Mcam	Melanoma cell adhesion molecule	0.37	0.29
ENSMUSG00000027469	Tpx2	TPX2microtubule-associated	0.37	0.33
ENSMUSG00000028678	Kif2c	Kinesin family member 2C	0.37	0.24
ENSMUSG00000027715	Ccna2	Cyclin A2	0.38	0.23
ENSMUSG00000048327	Ckap2l	Cytoskeleton associated protein 2-like	0.39	0.23
ENSMUSG00000040204	Pclaf	PCNA clamp associated factor	0.40	0.19
ENSMUSG00000029414	Kntc1	Kinetochore associated 1	0.42	0.24
ENSMUSG00000034311	Kif4	Kinesin family member 4	0.42	0.24
ENSMUSG00000031004	Mki67	Antigen identified by monoclonal antibody Ki 67	0.42	0.21
ENSMUSG00000020914	Top2a	Topoisomerase (DNA) II alpha	0.42	0.21
ENSMUSG00000033031	Cip2a	Cell proliferation regulating inhibitor of protein phosphatase 2A	0.42	0.32
ENSMUSG00000035783	Acta2	Actin alpha two smooth muscle aorta	0.43	0.48
ENSMUSG00000024795	Kif20b	Kinesin family member 20B	0.43	0.30
ENSMUSG00000038943	Prc1	Protein regulator of cytokinesis 1	0.43	0.26
ENSMUSG00000026494	Kif26b	Kinesin family member 26B	0.43	0.25
ENSMUSG00000023015	Racgap1	Rac GTPase-activating protein 1	0.43	0.26
ENSMUSG00000026605	Cenpf	Centromere protein F	0.44	0.25
ENSMUSG00000027306	Nusap1	Nucleolar and spindle associated protein 1	0.45	0.28
ENSMUSG00000028068	Iqgap3	IQ motif containing GTPase activating protein 3	0.46	0.21
ENSMUSG00000003779	Kif20a	Kinesin family member 20A	0.47	0.25
ENSMUSG00000005410	Mcm5	Minichromosome maintenance complex component 5	0.47	0.26
ENSMUSG00000034906	Ncaph	Non-SMC condensin I complex subunit H	0.47	0.27
ENSMUSG00000006398	Cdc20	Cell division cycle 20	0.48	0.29
ENSMUSG00000037313	Tacc3	Transforming acidic coiled-coil containing protein 3	0.48	0.36
ENSMUSG00000027699	Ect2	ect2 oncogene	0.48	0.26
ENSMUSG00000020330	Hmmr	Hyaluronan-mediated motility receptor (RHAMM)	0.50	0.28
ENSMUSG00000020649	Rrm2	Ribonucleotide reductase M2	0.50	0.26
ENSMUSG00000019942	Cdk1	Cyclin-dependent kinase 1	0.50	0.34
ENSMUSG00000024590	Lmnb1	Lamin B1	0.51	0.33
ENSMUSG00000037725	Ckap2	Cytoskeleton associated protein 2	0.55	0.42
Upregulated genes in Dyrk2^-/-^				
ID	GeneSymbol	Description	Ratio of Dyrk2^-/-^per wild-type in the presence of SAG	Ratio of Dyrk2^-/-^per wild-type in the absence of SAG
ENSMUSG00000056673	Kdm5d	Lysine (K)-specific demethylase 5D	Inf	Inf
ENSMUSG00000068457	Uty	Ubiquitously transcribed tetratricopeptide repeat gene Y chromosome	Inf	Inf
ENSMUSG00000069049	Ddx3y	DEAD (Asp-Glu-Ala-Asp) box polypeptide 3 Y-linked	Inf	8278
ENSMUSG00000069045	Eif2s3y	Eukaryotic translation initiation factor 2 subunit three structural gene Y-linked	Inf	Inf
ENSMUSG00000112616	Gm47434	Predicted gene 47434	719	Inf
ENSMUSG00000025582	Nptx1	Neuronal pentraxin 1	4.74	11.91
ENSMUSG00000024164	C3	Complement component 3	4.47	11.59
ENSMUSG00000039457	Ppl	Periplakin	4.30	11.11
ENSMUSG00000025784	Clec3b	C-type lectin domain family three member b	3.99	8.60
ENSMUSG00000002944	Cd36	CD36 molecule	3.20	3.45
ENSMUSG00000035385	Ccl2	Chemokine (C-C motif) ligand 2	2.86	2.84
ENSMUSG00000095478	Gm9824	Predicted pseudogene 9824	2.60	4.14
ENSMUSG00000038642	Ctss	Cathepsin S	2.58	3.19
ENSMUSG00000043719	Col6a6	Collagen type VI alpha 6	2.44	4.64
ENSMUSG00000033327	Tnxb	Tenascin XB	2.37	3.61
ENSMUSG00000069516	Lyz2	Lysozyme 2	2.30	3.08
ENSMUSG00000016494	Cd34	CD34 antigen	2.29	2.26
ENSMUSG00000042129	Rassf4	Ras association (RalGDS/AF-6) domain family member 4	2.29	3.43
ENSMUSG00000004730	Adgre1	Adhesion G-protein-coupled receptor E1	2.27	2.49
ENSMUSG00000030144	Clec4d	C-type lectin domain family member d	2.26	3.74
ENSMUSG00000029816	Gpnmb	Glycoprotein (transmembrane) nmb	2.22	2.66
ENSMUSG00000042286	Stab1	Stabilin 1	2.18	2.70
ENSMUSG00000020120	Plek	Pleckstrin	2.18	2.99
ENSMUSG00000040254	Sema3d	Sema domain immunoglobulin domain (Ig) short basic domain secreted (semaphorin) 3D	2.17	2.89
ENSMUSG00000005268	Prlr	Prolactin receptor	2.17	4.44
ENSMUSG00000024621	Csf1r	Colony-stimulating factor one receptor	2.10	2.74
ENSMUSG00000074896	Ifit3	Interferon-induced protein with tetratricopeptide repeats 3	2.04	3.96
ENSMUSG00000002985	Apoe	Apolipoprotein E	2.03	2.51
ENSMUSG00000057137	Tmem140	Transmembrane protein 140	2.02	3.18
ENSMUSG00000002289	Angptl4	Angiopoietin-like 4	2.02	5.94
ENSMUSG00000050335	Lgals3	Lectin galactose binding soluble 3	1.99	2.66
ENSMUSG00000090877	Hspa1b	Heat-shock protein 1B	1.98	2.13
ENSMUSG00000054404	Slfn5	Schlafen 5	1.96	3.77
ENSMUSG00000031209	Heph	Hephaestin	1.92	2.48
ENSMUSG00000027996	Sfrp2	Secreted frizzled-related protein 2	1.91	5.68
ENSMUSG00000050953	Gja1	Gap junction protein alpha 1	1.90	2.45
ENSMUSG00000005413	Hmox1	Heme oxygenase 1	1.90	1.97
ENSMUSG00000046805	Mpeg1	Macrophage expressed gene 1	1.85	2.57
ENSMUSG00000022037	Clu	Clusterin	1.83	3.06
ENSMUSG00000026389	Steap3	STEAP family member 3	1.81	2.24
ENSMUSG00000041577	Prelp	Proline arginine-rich end leucine-rich repeat	1.81	2.01
ENSMUSG00000027339	Rassf2	Ras association (RalGDS/AF-6) domain family member 2	1.80	2.72

These findings collectively support the potential mechanism that DYRK2 governs ciliogenesis by, at least in part, maintaining the expression of *Aurka* and other disassembly genes.

## Discussion

### DYRK2 is a positive regulator of Hh signaling

Although evidence indicates that DYRK2 plays important roles in the development of lower eukaryotes (Pellettieri et al., 2003; Pang et al., 2004; Lu and Mains, 2007), little is known regarding the functions of DYRK2 in mammalian development. In the present study, we demonstrate for the first time that DYRK2 is required for normal Hh signaling and embryogenesis in vivo. Varjosalo et al. have established a human full-length protein kinase cDNA and corresponding kinase activity-deficient mutant library, and they reported that DYRK2 functions as a negative regulator of Hh signaling via direct phosphorylation and induction of the proteasome-dependent degradation of GLIs using in vitro over-expression approaches (Varjosalo et al., 2008). In sharp contrast, our present study demonstrated using knockout approaches that endogenous protein levels of GLI2, and marginally the ratio of GLI3^REP^/GLI3^FL^, are decreased by deletion of *Dyrk2* in vitro after SAG-treatment. Consistently, *Dyrk2^-/-^* embryos exhibited some typical phenotypes of inactivation of Hh signaling in vivo as indicated by abnormal responses but not the elimination of ligands. Taken together with the observed loss of *Gli1* induction in *Dyrk2^-/-^* MEFs in vitro, we concluded that DYRK2 functions as a positive regulator of Hh signaling. GLI2 and GLI3, the key mediators of Hh signaling, are known to have specific and redundant functions (Mo et al., 1997). In skeletal patterning during development, *Dyrk2^-/-^* embryos exhibit phenotypes that are more similar to those of double *Gli2* and *Gli3* mutants than to those of each single mutant. Conversely, the deletion of other indispensable upstream Hh components such as *Smo* (Norman et al., 2009), *Ptch1 *(Svärd et al., 2006), *Gpr161 *(Hwang et al., 2018), and *SuFu *(Svärd et al., 2006) results in more severe defects that occur at earlier embryonic stages. The present data do not rule out the possibility that DYRK2 directly regulates Hh components. Despite this, given the evidence that *Dyrk2^-/-^* embryos and cells possess morphological abnormalities in primary cilia, it is clear that DYRK2 plays a pivotal role in regulating Hh signaling via the control of ciliogenesis.

### *Dyrk2* is a novel ciliogenesis-related gene in mice

Intriguingly, we found that DYRK2 negatively regulates ciliogenesis. In *Chlamydomonas*, certain mutations that cause flagella to assemble to excessive length (i.e. negatively regulator of ciliogenesis) have been identified, and these include mutations in LF1 through LF5 (Wilson et al., 2008; Tam et al., 2013). Among these, LF2, LF4, and LF5 encode protein kinases and are homologs of vertebrate CCRK, MAK/ICK/MOK, and CDKL5, respectively. In addition to these genes, GSK3β also negatively regulates cilia and flagella length (Yuan et al., 2012). Interestingly, DYRK2 belongs to the same kinase group as CCRK, MAK/ICK/MOK, CDKL5, and GSK3β (the CMGC group) (Becker and Sippl, 2011). To the best of our knowledge, however, the present study demonstrates for the first time that DYRK2 is a ciliogenesis-related gene and a negative regulator of ciliogenesis. Among these kinases, *Dyrk2^-/-^* embryos exhibit similar phenotypes to *Ick*-deletion mice in vivo such as skeletal defects and cilia morphology, although they do not possess a hydrocephalus defect (Moon et al., 2014). In response to *Ick-* or *Mok-*knockdown, cilia length depends on the activation of mTORC1 signaling (Bolton et al., 2007). Our previous study reported the activation of mTORC1 signaling in *DYRK2-*knockdown human breast cancer cells (Mimoto et al., 2017). As expected, mTORC1 signaling was slightly activated in *Dyrk2^-/-^* MEFs; however, rapamycin, an inhibitor of mTORC1, did not significantly affect cilia length in *Dyrk2^-/-^* MEFs. Thus, DYRK2 may control ciliogenesis through different mechanisms than those of other CMGC-kinases.

### DYRK2 is required for ciliogenesis and the dynamic trafficking of Hedgehog components in cilia

**Figure 10.**
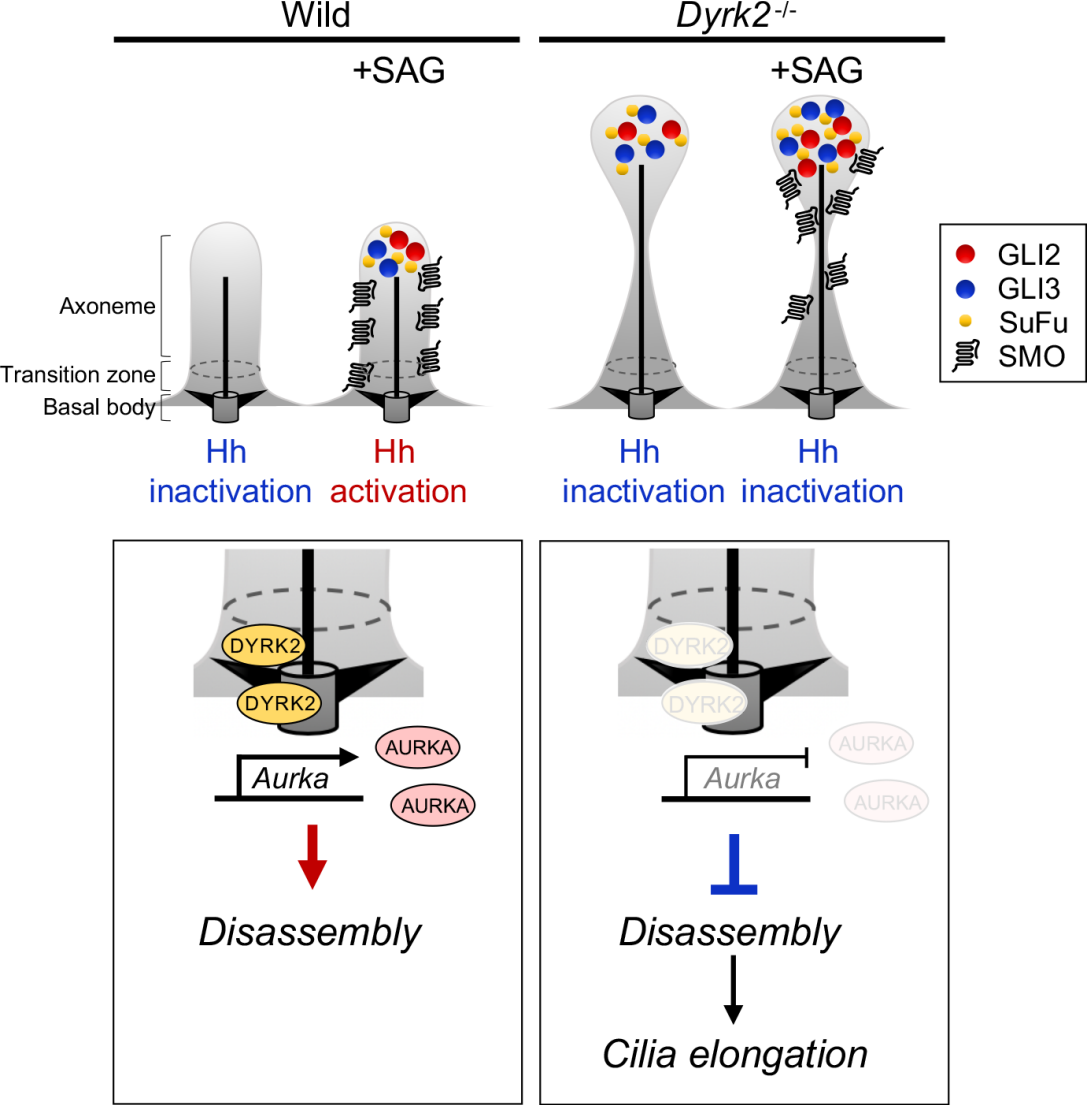
Schematic representation of DYRK2 in ciliogenesis and Hh signaling. (Left panel) A schematic model of normal ciliogenesis and response to stimulation with Hh ligand. (Right panel) A schematic model ciliogenesis and response to stimulation with Hh ligand in *Dyrk2*-deletion. The morphology of primary cilia in *Dyrk2^-/-^* MEFs was elongated and often bulged at the tips. In *Dyrk2^-/-^* cells, downregulation of *Aurka* and other ciliary disassembly genes caused suppression of disassembly and elongation of primary cilia. Furthermore, abnormal ciliary trafficking caused accumulation of GLI2, GLI3, and SuFu in *Dyrk2^-/-^* cells. Consequently, the induction of Hh signaling is drastically suppressed by deletion of *Dyrk2.*

Abnormal ciliary trafficking of Hh components causes dysfunction in Hh signaling in several types of mutant mice (He et al., 2014; Moon et al., 2014). *Dyrk2^-/-^* MEFs and embryos possessed disordered accumulation of Hh components (GLI2, GLI3, and SuFu) at cilia tips and exhibited elongation of cilia. On the other hand, SMO recruitment dependent on ligand stimulation was normally observed in *Dyrk2^-/-^* MEFs (Figure 10). Moreover, DYRK2 localizes at TZ, which acts as a selective barrier to control ciliary import and export of proteins (Gerhardt et al., 2016). Given the findings that ciliary localization and ciliary disorders were observed in *Dyrk2^-/-^* cells, DYRK2 could be involved in regulation of ciliary protein entry and exit. The functions of DYRK2 at TZ, however, remains to be elucidated.

As a first step to elucidate the functions of DYRK2 in cilia, we focused on factors involved in ciliary length control using a transcriptome approach, and we identified the downregulation of genes related to ciliary resorption mechanisms for proliferation in *Dyrk2^-/-^* MEFs, such as *Aurka*, *Plk1*, *Ube2c*, *Tpx2, Kif2c,* and *Cdc20*. Among these, AURKA has been well-characterized as a disassembly factor, as transient knockdown by siRNA or treatment with an inhibitor of AURKA completely blocked ciliary disassembly during proliferation (Pugacheva et al., 2007; Inoko et al., 2012). In contrast to ciliary disassembly under proliferation, the function of AURKA for controlling ciliary length at steady state is unclear. In the present study, transient knockdown of *Aurka* under serum-starvation conditions induced elongation of cilia in a manner similar to that observed in *Dyrk2^-/-^* MEFs. These data imply that the down-regulation of *Aurka* is, at least in part, associated with the phenotypes observed after deletion of *Dyrk2.* The expression of *Aurka* is known to be regulated by pathways such as YAP/TAZ (Kim et al., 2015a) and AKT signaling (Plotnikova et al., 2015). The molecular mechanisms underlying DYRK2-mediated *Aurka* regulation and ciliary trafficking remain unclear. Further research is required to elucidate these mechanisms.

### A possible relationship between DYRK2 and human ciliopathy

A number of syndromes caused by disorders involving ciliary proteins are categorized as skeletal ciliopathies, and these include short-rib thoracic dysplasia (SRTD), Jeune asphyxiating thoracic dystrophy (JATD), orofaciodigital syndrome (OFD), Ellis-van Creveld syndrome (EVC), and cranioectodermal dysplasia (CED) (Reiter and Leroux, 2017). In mice, deletion of *Dyrk2* induces morphological abnormalities in primary cilia and skeletal defects in vivo. Additionally, DYRK2 is a ciliary protein that is primarily localized at the basal body and the TZ, which contains a growing number of ciliopathy proteins (Reiter et al., 2012). While the present study does not include any evidence to support the relationship between DYRK2 and human disease, our results do suggest a possibility that *DYRK2* is involved in human ciliopathy, particularly in regard to skeletal disorders. Further investigations involving exome sequencing or genome-wide association studies using human patients will prove useful to clarify this issue.

### Conclusion

In summary, we identified DYRK2 as a novel mammalian ciliogenesis-related gene in vivo and in vitro. Deletion of *Dyrk2* induces abnormal ciliary morphology and trafficking of Hh pathway components and suppresses Hh signaling during mouse embryogenesis. The abnormal ciliogenesis in *Dyrk2^-/-^* cells is partially caused by downregulation of *Aurka* and other disassembly genes. These findings will allow for a more complete understanding of the molecular mechanisms underlying embryogenesis, ciliogenesis, and human ciliopathy.

## Materials and methods

**Key resources table:** 

Reagent type (species) or resource	Designation	Source or reference	Identifiers	Additional information
Genetic reagent (*M. musculus*)	*Dyrk2*^-/-^ mouse	This paper	N/A	Maintained in K. Yoshida lab.
Cell line (*M. musculus*)	Wild-type and *Dyrk2*^-/-^ MEFs	This paper	N/A	Maintained in K. Yoshida lab.
Cell line (*H. sapiens*)	hTERT-RPE1	ATCC	Cat# CRL-4000 RRID:CVCL_4388	
Transfected construct (*M. musculus*)	mouse *Aurka*/pEGFP-C1	This paper	N/A	See Materials and methods subsection ‘Plasmid constructs’
Transfected construct (*M. musculus*)	mouse *Dyrk2*/FN22K-Halo Tag-CMVd1-Flexi-vector	This paper	N/A	See Materials and methods subsection ‘Plasmid constructs’
Transfected construct (*M. musculus*)	*Dyrk2* targeting vector	Knockout Mouse Project Repository	PG00105_X_1_G09, PG00105_X_1_E04	See Materials and methods subsection ‘Plasmid constructs’
Recombinant DNA regent	Plasmid pEGFP-C1 (empty vector)	TaKaRa Bio	Cat# 6084–1	
Recombinant DNA regent	Plasmid pFN22K-Halo Tag-CMVd1-Flexi-vector (empty vector)	Promega	Cat# G2851	
Transfected construct (*M. musculus*)	*Dyrk2* targeting vector	Knockout Mouse Project Repository	PG00105_X_1_G09, PG00105_X_1_E04	
Biological sample (Adenovirus)	Adenovirus-*Cre*	Yokoyama-Mashima et al., 2019 doi: 10.1016/j.canlet.2019.02.046.	N/A	
Biological sample (Adenovirus)	Adenovirus-human *DYRK2*	Yokoyama-Mashima et al., 2019 doi: 10.1016/j.canlet.2019.02.046.	N/A	
Biological sample (Adenovirus)	Adenovirus-human *DYRK2-K251R*	Yokoyama-Mashima et al., 2019 doi: 10.1016/j.canlet.2019.02.046.	N/A	
Biological sample (Adenovirus)	Adenovirus-GFP	Yokoyama-Mashima et al., 2019 doi: 10.1016/j.canlet.2019.02.046.	N/A	
Antibody	Anti-Acetylated-tubulin (Mouse monoclonal)	Sigma-Aldrich	Cat# T7451, RRID:AB_609894	ICC (1:2000)
Antibody	Anti-ARL13B (Mouse monoclonal)	Abcam	Cat# ab136648,N/A	ICC (1:300)
Antibody	Anti-ARL13B (Rabbit polyclonal)	Proteintech	Cat# 17711–1-AP, RRID:AB_2060867	ICC (1:400)
				IHC (1:400)
Antibody	Anti-AURKA (Mouse monoclonal)	BD Transduction	Cat# 610938, RRID:AB_398251	WB (1:1000)
Antibody	Anti-DYRK2 (Rabbit polyclonal)	Sigma-Aldrich	Cat# HPA027230, RRID:AB_1847925	WB (1:1000)
				ICC (1:400)
Antibody	Anti-FOXA2 (Mouse monoclonal)	Developmental Studies Hybridoma Bank	Cat# 4C7, RRID:AB_528207	IHC (1:8)
Antibody	Anti-CP110 (Rabbit polyclonal)	Proteintech	Cat# 12780–1-AP, RRID:AB_10638480	WB (1:1000)
Antibody	Anti-GAPDH (Mouse monoclonal)	Santa Cruz Biotechnology	Cat# sc-32233, RRID:AB_627679	WB (1:3000)
Antibody	Anti-GFP (Chicken polyclonal IgY)	Aves Labs	Cat# GFP-1020, RRID:AB_10000240	ICC (1:500)
Antibody	Anti-GFP (Rabbit monoclonal)	Abcam	Cat# ab183734, RRID:AB_2732027	WB (1:30000)
Antibody	Anti-GLI1 (Rabbit polyclonal)	Cell Signaling Technology	Cat# 2534, RRID:AB_2294745	WB (1:500)
				ICC (1:100)
Antibody	Anti-GLI2 (Goat polyclonal)	R and D systems	Cat# AF3635, RRID:AB_2111902	WB (1:500)
				ICC (1:50)
				IHC (1:50)
Antibody	Anti-GLI3 (Goat polyclonal)	R and D systems	Cat# AF3690, RRID:AB_2232499	WB (1:200)
				ICC (1:100)
				IHC (1:150)
Antibody	Anti-gamma-tubulin (Goat polyclonal)	Santa Cruz Biotechnology	Cat# sc-7396, RRID:AB_2211262	ICC (1:3500)
Antibody	Anti-gamma-tubulin (Mouse monoclonal)	Santa Cruz Biotechnology	Cat# sc-17787, RRID:AB_628417	ICC (1:400)
				IHC (1:400)
Antibody	Anti-HaloTag (Rabbit polyclonal)	Promega	Cat# G9281, RRID:AB_713650	ICC (1:700)
Antibody	Anti-IFT140 (Rabbit polyclonal)	Proteintech	Cat# 17460–1-AP, RRID:AB_2295648	ICC (1:100)
Antibody	Anti-IFT81 (Rabbit polyclonal)	Proteintech	Cat# 11744–1-AP, RRID:AB_2121966	ICC (1:50)
Antibody	Anti-IFT88 (Rabbit polyclonal)	Proteintech	Cat# 13967–1-AP, RRID:AB_2121979	ICC (1:100)
Antibody	Anti-KATANIN p60 (Mouse monoclonal)	Santa Cruz Biotechnology	Cat# sc-373814, RRID:AB_11014191	WB (1:1000)
Antibody	Anti-KI67 (Rabbit monoclonal)	Abcam	Cat# ab16667, RRID:AB_302459	ICC (1:500)
Antibody	Anti-NPHP1 (Mouse monoclonal)	SIGMA-Aldrich	Cat# MABS2185,N/A	ICC (1:100)
Antibody	Anti-mTORC1 (Rabbit monoclonal)	Cell Signaling Technology	Cat# 2972, RRID:AB_330978	WB (1:1000)
Antibody	Anti-NKX2.2 (Mouse monoclonal)	Developmental Studies Hybridoma Bank	Cat# 74.5A5, RRID:AB_531794	IHC (1:10)
Antibody	Anti-NKX6.1 (Mouse monoclonal)	Developmental Studies Hybridoma Bank	Cat# F55A10, RRID:AB_532378	IHC (1:100)
Antibody	Anti-OLIG2 (Rabbit monoclonal)	abcam	Cat# ab109186, RRID:AB_10861310	IHC (1:500)
Antibody	Anti-PAX6 (Mouse monoclonal)	Santa Cruz Biotechnology	Cat# sc-81649, RRID:AB_1127044	IHC (1:400)
Antibody	Anti-Phosho-S6 (Ser 235/236) (Rabbit monoclonal)	Cell Signaling Technology	Cat# 2211, RRID:AB_331679	WB (1:2000)
Antibody	Anti-P-4EBP1(Thr 37/46) (Rabbit monoclonal)	Cell Signaling Technology	Cat# 2855, RRID:AB_560835	WB (1:1500)
Antibody	Anti-SMO (Mouse monoclonal)	Santa Cruz Biotechnology	Cat# sc-166685, RRID:AB_2239686	ICC (1:100)
Antibody	Anti-SuFu (Mouse monoclonal)	Santa Cruz Biotechnology	Cat# sc-137014, RRID:AB_2197315	ICC (1:100)
Antibody	Anti-S6 (Rabbit monoclonal)	Cell Signaling Technology	Cat# 2217, RRID:AB_331355	WB (1:2000)
Antibody	Anti-4EBP1 (Rabbit monoclonal)	Cell Signaling Technology	Cat# 9644, RRID:AB_2097841	WB (1:3000)
Sequence-based reagent	Human *DYRK2* siRNA#1	BEX	608481	
Sequence-based reagent	Human *DYRK2* siRNA#2	ThermoFisher Scientific	HSS112284	
Sequence-based reagent	Mouse *Dyrk2* siRNA#1	ThermoFisher Scientific	4390771 (s87545)	
Sequence-based reagent	Mouse *Dyrk2* siRNA#2	ThermoFisher Scientific	4390771 (s87546)	
Sequence-based reagent	Mouse *Aurka* siRNA#1	Integrated DNA Technologies	mm.Ri.Aurka.13.1	
Sequence-based reagent	Mouse *Aurka* siRNA#2	Integrated DNA Technologies	mm.Ri.Aurka.13.4	
Sequence-based reagent	Mouse *Cdc20* siRNA	Integrated DNA Technologies	mm.Ri.Cdc20.13.2	
Sequence-based reagent	Mouse *Kif2c* siRNA	Integrated DNA Technologies	mm.Ri.Kif2c.13.3	
Sequence-based reagent	Mouse *Plk1* siRNA	Integrated DNA Technologies	mm.Ri.Plk1.13.1	
Sequence-based reagent	Mouse *Tpx2* siRNA	Integrated DNA Technologies	mm.Ri.Tpx2.13.1	
Sequence-based reagent	Mouse *Ube2c* siRNA	Integrated DNA Technologies	mm.Ri.Ube2c.13.1	
Sequence-based reagent	Negative Control DsiRNA (siNegative)	Integrated DNA Technologies	51-01-14	
Sequence-based reagent	Silencer Select Negative Control (siControl)	ThermoFisher Scientific	4390843	
Chemical compound, drug	InSolution SAG	Merck	566660	
Chemical compound, drug	Rapamycin	LC Laboratories	R-5000	
Software, algorithm	BZ-X800 Analyzer	Keyence	BZ-X800 Analyzer	
Software, algorithm	Excel	Microsoft	Mac2019	
Software, algorithm	Fusion	M and S Instruments	Fusion	
Software, algorithm	GraphPad Prism 7	GraphPad Software Inc	Mac OS X	
Software, algorithm	PikoReal Software 2.1	ThermoFisher Scientific	PikoReal Software 2.1	

### Generation of *Dyrk2* knockout mice (*Dyrk2^-/-^*)

*Dyrk2^-/-^* mice were generated using the knockout-first strategy (Skarnes et al., 2011). A schematic representation of the targeted *Dyrk2* allele is provided in Figure 1—figure supplement 1A. The *Dyrk2* targeting vector (PG00105_X_1_G09 and PG00105_X_1_E04) was obtained from the Knockout Mouse Project Repository (*Dyrk2* targeting project: 337–66440). Gene-targeting methods were performed according to standard protocols. Briefly, linearized vectors were electroporated into JM8A3.N1 embryonic stem (ES) cells. G418-resistant ES cell clones were analyzed using Southern blot analysis for the presence of the correct targeted-allele using BglII digestion and a 3’ external probe. Hybridization with the 3’ external probe detected 10.7 kb (wild-type allele) and 17.0 kb (targeted tm1a allele) BglII bands (Figure 1—figure supplement 1A). Six positive ES clones out of 240 clones were obtained. Chimeric mice were created by injection of the targeted ES cells into C57BL/6J blastocysts and were mated with C57BL/6J WT mice to establish germline-transmitted founders. Heterozygous knockout-first (*Dyrk2*^tm1a^) mice were identified using Southern blotting. An exon three knockout allele (*Dyrk2*^tm1b^) was generated by mating the *Dyrk2*^tm1a^ mice the with CAG-Cre mice (Figure 1—figure supplement 1A). For genotyping and validation of knockout alleles, we performed PCR using the primers listed in Table 2.

**Table 2.** List of primer sets.

For genotyping			
Gene	Sequence (5'→3')		Accession number
*Dyrk2 tm1b-WT*	Forward	TGGGTCCAAATGCAAAGAAACGCCA	NC_000076.6
	Reverse	GCTTCTCGTTCCGCACCATCTTCAG	
*Dyrk2 tm1b-KO*	Forward	CCTTCTCCCTCCTCCACTCTGACCCA	NC_000076.6
	Reverse	CCACACCTCCCCCTGAACCTGAAAC	
For amplification of the probes for in situ hybridization or Southern blotting			
Gene	Sequence (5'→3')		Accession number
Mouse *Foxf2*	Forward	GAGATTAACCCTCACTAAAGGGAGGTTATGGTGGCCTCGACAT	NM_010225.2
	Reverse	GAGTAATACGACTCACTATAGGGACACACACACCTCCCTTTTCA	
Mouse *Gli1*	Forward	GAGTATTTAGGTGACACTATAGAAGCAGGGAAGAGAGCAGACTG	NM_010296.2
	Reverse	GAGTAATACGACTCACTATAGGGGCTGAGTGTTGTCCAGGTC	
Mouse *Ptch1*	Forward	GAGATTAACCCTCACTAAAGGGACATGGCCTCGGCTGGTAAC	NM_008957.3
	Reverse	GAGTAATACGACTCACTATAGGGTGTACCCATGGCCAACTTCG	
Southern for *Dyrk2*	Forward	CTTCGAATCCTTTTATCCTTCAGGC	NC_000076.6
	Reverse	ACATCATGTTCATTGGTTTTGCTCT	
For cloning			
Gene	Sequence (5'→3')		Accession number
Mouse *Aurka CDS*	Forward	GGACTCAGATCTCGAGACATGGCTGTTGAGGGCG	NM_011497.4
	Reverse	GTCGACTGCAGAATTCCTAAGATGATTTGCTGGTTG	
Mouse *Dyrk2 CDS*	Forward	GTGCGCGATCGCCATGTTAACCAGGAAACCTTCGGC	NM_001014390.2
	Reverse	CTCCGTTTAAACGCTAACGAGTTTCGGCAACAC	
For real-time PCR			
Gene	Sequence (5'→3')		Accession number
Human *DYRK2*	Forward	GGGGAGAAAACGTCAGTGAA	NM_006482.3
	Reverse	TCTGCGCCAAATTAGTCCTC	
Human *HPRT1*	Forward	GGACTAATTATGGACAGGACTG	NM_000194.3
	Reverse	GCTCTTCAGTCTGATAAAATCTAC	
Mouse *Aurka*	Forward	CACACGTACCAGGAGACTTACAGA	NM_011497.4
	Reverse	AGTCTTGAAATGAGGTCCCTGGCT	
Mouse *Cdc20*	Forward	GAGCTCAAAGGACACACAGC	NM_023223.2
	Reverse	GCCACAACCGTAGAGTCTCA	
Mouse *Dyrk2*	Forward	CTACCACTACAGCCCACACG	NM_001014390.2
	Reverse	TCTGTCCGTGGCTGTTGA	
Mouse *Foxf2*	Forward	AGCATGTCTTCCTACTCGTTG	NM_010225.2
	Reverse	TCTTTCCTGTCGCACACT	
Mouse *Gli1*	Forward	GCACCACATCAACAGTGAGC	NM_010296.2
	Reverse	GCGTCTTGAGGTTTTCAAGG	
Mouse *Hprt*	Forward	CTCATGGACTGATTATGGACAGGAC	NM_013556.2
	Reverse	GCAGGTCAGCAAAGAACTTATAGCC	
Mouse *Kif2c*	Forward	GAGAGCAAGCTGACCCAGG	NM_134471.4
	Reverse	CCTGGTGAGATCATGGCGATC	
Mouse *Plk1*	Forward	CCAAGCACATCAACCCAGTG	NM_011121.4
	Reverse	TGAGGCAGGTAATAGGGAGACG	
Mouse *Ptch1*	Forward	CTCTGGAGCAGATTTCCAAGG	NM_008957.3
	Reverse	TGCCGCAGTTCTTTTGAATG	
Mouse *Shh*	Forward	GTGAAGCTGCGAGTGACCG	NM_009170.3
	Reverse	CCTGGTCGTCAGCCGCCAGCACGC	
Mouse *Tpx2*	Forward	GCGAGGTTGTCAGGTGTGTA	NM_001141977.1
	Reverse	TTGATAAAGTCGGTGGGGGC	
Mouse *Ube2c*	Forward	CTGCTAGGAGAACCCAACATC	NM_026785.2
	Reverse	GCTGGAGACCTGCTTTGAATA	

### Animal care

Mice were housed individually in a temperature-controlled room under a 12 hr light/dark cycle. Determination of pregnancy in mice was achieved by the observation of a vaginal plug on day 0.5 of gestation. Animals were euthanized by anesthesia. The animal experiment protocol was approved by the Institutional Animal Care and Use Committee of Jikei University (No. 2017–065 and 2018–031), and the studies were performed in accordance with the Guidelines for the Proper Conduct of Animal Experiments of the Science Council of Japan.

### Alcian blue and alizarin red staining

Euthanized wild-type and *Dyrk2^-/-^* mice at E18.5 and E16.5 were skinned, eviscerated, and fixed in 100% EtOH. For skeletal analysis, the embryos were stained with 1% Alcian Blue (Wako Pure Chemicals, Osaka, Japan) dissolved in 20% glacial acetic acid and 80% EtOH and 0.01% Alizarin Red (Sigma-Aldrich, St. Louis, MO) dissolved in 1% KOH. The excised tissues were observed using a stereo microscope (BioTools, Gunma, Japan). Ten embryos of each wild-type and *Dyrk2^-/-^* mice were analyzed.

### In situ hybridization

In situ hybridization was performed according to a previous report (Fujiwara et al., 2007). Briefly, each digoxigenin (DIG)-labeled cRNA probe was amplified by PCR using primer sets (Table 2) and labeled using the Roche DIG RNA labeling kit (Merck, Schwalbach, Germany). Embryos at E10.5 and the heads of mice at E14.5 were fixed using MEMFA (2 mM EGTA, 1 mM MgSO4, and 3.7% formaldehyde) in 100 mM MOPS (pH 7.5) overnight at 4°C, and this was followed by immersion in 30% trehalose (Wako) in 20 mM HEPES to cryoprotect the tissues. Cryosections (7 μm thickness) from the transverse or sagittal plane were hybridized with DIG-labeled cRNA probe and were visualized with alkaline phosphatase-conjugated anti-DIG antibody (Merck) using 4-nitroblue tetrazolium chloride (NBT; Merck) and 5-bromo-4-chloro-3-indolyl phosphate (BCIP; Merck). The sections were observed under a BZ-X800 microscope (KEYENCE, Osaka, Japan).

### Immunohistochemistry and hematoxylin and eosin (HE)-staining

Embryos at E10.5, E13.5, and E18.5 were fixed and sliced as described above. Depending on the antibody, the sections were antigen retrieved by an ImmunoSaver (Nisshin EM, Tokyo, Japan) for 60 min at 80°C. The sections were incubated with 10% (v/v) fetal bovine serum and 0.4% (v/v) Triton X-100 in HEPES buffer (blocking buffer). After washing, the sections were incubated with primary antibodies (Key resources table) in blocking buffer at 4°C overnight. After the immunoreaction, the sections were incubated with secondary antibodies using Cy3-, Cy5-, or FITC-conjugated AffiniPure donkey anti-goat, rabbit, and mouse IgG (Jackson ImmunoResearch, West Grove, PA). The sections were washed and incubated in VECTASHIELD Mounting Medium (Vector Laboratories, Burlingame, CA) containing 4,6′-diamidino-2-phenylindole dihydrochloride (DAPI). For HE-staining, the sections were stained using standard procedures. The sections were observed under a BZ-X800 fluorescence microscope (KEYENCE).

### Scanning electron microscopy (SEM)

Wild-type and *Dyrk2^-/-^* embryos at E10.5 were washed with 0.1 M phosphate buffer (PB) (pH7.5) and fixed with 2% glutaraldehyde (TAAB Laboratories Equipment, Berkshire, England) in 0.1 M PB (pH 7.4) for 1 week at 4°C. The embryos were placed in tannic acid in 0.1 M PB for 2 hr at room temperature in darkness, and then immersed in 1% OsO_4_ solution for 2 hr at room temperature. After dehydration in graded ethanol, the samples were transferred into isoamyl acetate and dried at the critical point in liquid CO_2_, and this was followed by a metal coating procedure (Hitachi, Tokyo, Japan). The surfaces of tissues were then observed using scanning electron microscopy (Hitachi).

### Plasmid constructs

Full-length cDNA fragments of mouse *Dyrk2* and *Aurka* were amplified by PCR and cloned in frame into the pFN22K-HaloTag-CMVd1-Flexi-vector (Promega, Madison, WI) and pEGFP-C1 (TaKaRa Bio, Otsu, Japan), respectively. The nucleotide sequences of the primers used are listed in Table 2.

### Cell culture and transfection

Primary mouse embryonic fibroblast (MEFs) were generated from wild-type and *Dyrk2^-/-^* embryos at E13.5. MEFs and immortalized human retinal pigment epithelia cells (hTERT-RPE1; Cat# CRL-4000, RRID:CVCL_4388, ATCC, Manassas, VA) were cultured in DMEM (nacalai tesque, Kyoto, Japan) with 10% FBS (biowest, Nuaille, France), 1% GultaMAX (Gibco, Gaithersburg, MD), and 1% Penicillin-streptomycin (nacalai tesque) at 37°C under 5% CO_2_. hTERT-RPE1 cells were authenticated by the STR profiling and negative for mycoplasma contamination. To induce ciliogenesis, cells were grown to 80–90% confluency and serum-starved (0.5% FBS) for 24 hr. For SAG-stimulation, cells were treated with 100 nM SAG (Merck) for 24 hr after serum-starvation. For rapamycin-stimulation, cells were treated with 0.5 µM rapamycin (LC Laboratories, Woburn, MA) for 24 hr after serum-starvation. Transient knockdown was achieved using the Lipofectamine RNAiMAX transfection regent (ThermoFisher Scientific, Waltham, MA) for 48 hr under serum-starvation conditions according to the manufacturer’s instructions with a final concentration of 6–20 nM siRNA (Key resources table). For over-expression of DYRK2-HaloTag, transfection was performed using X-tremeGENE9 (Merck) for hTERT-RPE1 cells according to the manufacturer’s instructions and the cells were cultured for 24 hr under serum-starvation condition for ciliogenesis. For over-expression of AURKA-EGFP or EGFP, transfection was performed using Xfect (TaKaRa Bio) for MEFs according to the manufacturer’s instructions, and the cells were cultured for 24 hr under serum-starvation condition for ciliogenesis.

### Adenovirus infection

Adenovirus construction and infections were performed according to a previous report (Maekawa et al., 2013; Yokoyama-Mashima et al., 2019). Briefly, Flag-DYRK2 and Flag-DYRK2-K251R (Taira et al., 2010; Mimoto et al., 2013) were expressed depending upon *Cre*-expression. Following infection at a MOI (multiplicity of infection) of 100, MEFs were extracted for gene-expression analysis at 60 hr post-infection. MOI for MEFs was determined using an adenovirus construct for GFP-expression.

### Immunoblotting

Tissues (the limb buds at E13.5) and MEFs were washed in twice and lysed using RIPA buffer containing several inhibitors (1 mM PMSF, 10 µg/ml Aprotinin, 1 µg/ml Leupeptin, 1 µg/ml Pepstatin A, 1 mM Na_3_VO_4_, 10 mM NaF, and 1 mM DTT). Equal amounts of protein (5 µg) were resolved on 4–15% Mini-PROTEA TGX Precast Protein Gels (BioRad, Hercules, CA). After electrophoresis, proteins were transferred to PVDF membranes (Merck). Membranes were blocked with 5% skim milk in tris-buffered saline (TBS) containing 0.05% Tween 20 (TBST) or 0.1% casein/gelatin in TBST, depending on the antibody. Primary and secondary antibodies were reacted in each blocking buffer (Key resources table). Signals were detected using a chemiluminescent regent, ImmunoStar LD (Wako). Signals were observed and band intensity was measured using a Fusion-Solo system (M and S Instruments, Tokyo, Japan).

### Quantitative real-time polymerase chain reaction (qPCR)

Total RNAs were prepared from tissues (the limbs of E13.5, the mandibular arches of E10.5, and whole embryos at E9.5) and MEFs using the RNeasy mini kit (QIAGEN, Germantown, MD) or ISOGEN II (Nippon Gene, Tokyo, Japan), respectively. Reverse transcripts were obtained using PrimeScript Reverse Transcriptase (TaKaRa Bio) and subjected to qPCR using a PIKOREAL96 system (ThermoFisher Scientific). Reactions were performed in KAPA SYBR FAST qPCR Master Mix (NIPPON Genetics, Tokyo, Japan) that included 0.2 μM of a specific primer set for each gene (Table 2). Data were calculated by the comparative C_T_ method (ΔC_T_ method) to estimate the mRNA copy number relative to that of *Hprt* as an internal standard. The DNA sequence of the PCR product was confirmed by nucleotide sequencing (data not shown).

### Immunocytochemistry

For immunocytochemistry, MEFs and hTERT-RPE1 cells were cultured on 8-well chamber slides (ThermoFisher Scientific) coated with Poly-D-lysin (Sigma-Aldrich). Cells were fixed and antigen retrieved depending on the antibody. The primary antibody reaction was performed at an appropriate dilution (Key resources table) in the presence of blocking buffer at 4°C overnight. After immunoreactions, cells were incubated with secondary antibodies using Cy3-, Cy5-, or FITC-conjugated AffiniPure donkey anti-goat, rabbit, and mouse IgG, and chicken IgY (Jackson ImmunoResearch). The cells were then washed and incubated with DAPI. Immunofluorescence was observed under a BZ-X800 fluorescence microscope (KEYENCE).

### RNA-Seq

Total RNAs were prepared from MEFs cultured in the absence or presence of 100 nM SAG for 24 hr using RNeasy mini kit (QIAGEN) with DNase I treatment (QIAGEN). Materials were enriched for polyA sequences, and quantitative RNA-sequencing was performed using an Illumina HiSeq (Illumina, San Diego, CA). Cutadapt v1.9.1 was used to trim and filter reads, and clean data were aligned to the reference genome (ENSEMBLE, GRCm38.97) using the software HISAT 2 (v2.0.1). Relative gene expression was quantified and normalized in a FPKM (fragments per kilobase of transcript per million mapped reads) format.

### STRING and gene ontology (GO) analysis

To determine the presence of interactions/partnerships among downregulated genes in *Dyrk2^-/-^* MEFs, protein-protein interaction networks were extracted from the STRING database (https://string-db.org) and drawn by STRING v11. Gene ontology (GO) analysis was also performed using STRING v11 to demonstrate the biological processes enriched in the altered genes. The resulting GO terms that possessed a false discovery rate (FDR) of less than 0.005 were considered as enriched biological processes.

### Statistical analysis

Each experiment was confirmed by at least three independent biological replicates per condition. Data are presented as the means ± SEM. Prism seven software (GraphPad, San Diego, CA, USA) was used for statistical analyses. Means between two groups were compared using the Student’s *t*-test. Multiple inter-group differences were analyzed by one-way ANOVA (analysis of variance) followed by Tukey’s multiple comparison test.

## Data Availability

Data except for RNA-seq in this study are included in the manuscript and supporting files. Source data files have been provided: Figure 2-source data 1 Figure 2-figure supplement 1-source data 1 Figure 3-source data 1 Figure 3-source data 2 Figure 3-figure supplement 1-source data 1 Figure 3-figure supplement 2-source data 1 Figure 4-source data 1 Figure 4-figure supplement 1-source data 1 Figure 4-figure supplement 2-source data 1 Figure 4-figure supplement 3-source data 1 Figure 6-source data 1 Figure 6-figure supplement 3-source data 1 Figure 7-source data 1 Figure 8-source data 1 Figure 9-source data 1. RNA-seq data have been deposited in Dryad under accession code URL https://doi.org/10.5061/dryad.pnvx0k6j8. The following dataset was generated: YoshidaSAokiKFujiwaraKNakakuraTKawamuraAYamadaKOnoMYogosawaSYoshidaK2020The novel ciliogenesis regulator DYRK2 governs Hedgehog signaling during mouse embryogenesisDryad Digital Repository10.5061/dryad.pnvx0k6j8PMC741048932758357

## Additional files

Transparent reporting form
